# Profile‐likelihood Bayesian model averaging for two‐sample summary data Mendelian randomization in the presence of horizontal pleiotropy

**DOI:** 10.1002/sim.9320

**Published:** 2022-01-20

**Authors:** Chin Yang Shapland, Qingyuan Zhao, Jack Bowden

**Affiliations:** ^1^ MRC Integrative Epidemiology Unit University of Bristol Bristol UK; ^2^ Population Health Sciences University of Bristol Bristol UK; ^3^ Department of Pure Mathematics and Mathematical Statistics University of Cambridge Cambridge UK; ^4^ College of Medicine and Health University of Exeter Exeter UK

**Keywords:** Bayesian model averaging, horizontal pleiotropy, InSIDE violation, two‐sample summary data Mendelian randomization, weak instruments

## Abstract

Two‐sample summary data Mendelian randomization is a popular method for assessing causality in epidemiology, by using genetic variants as instrumental variables. If genes exert pleiotropic effects on the outcome not entirely through the exposure of interest, this can lead to heterogeneous and (potentially) biased estimates of causal effect. We investigate the use of Bayesian model averaging to preferentially search the space of models with the highest posterior likelihood. We develop a Metropolis‐Hasting algorithm to perform the search using the recently developed MR‐RAPS as the basis for defining a posterior distribution that efficiently accounts for pleiotropic and weak instrument bias. We demonstrate how our general modeling approach can be extended from a standard one‐component causal model to a two‐component model, which allows a large proportion of SNPs to violate the InSIDE assumption. We use Monte Carlo simulations to illustrate our methods and compare it to several related approaches. We finish by applying our approach to investigate the causal role of cholesterol on the development age‐related macular degeneration.

## 1 INTRODUCTION

**FIGURE 1:**
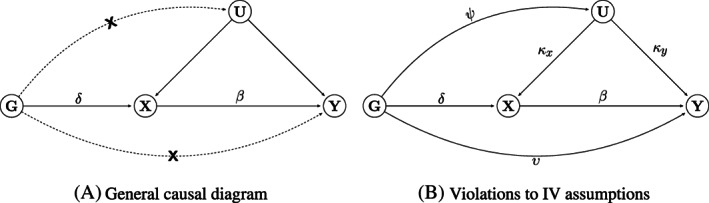
Causal diagrams representing the hypothesized relationship between genetic instrument (*G*), exposure (*X*), outcome (*Y*), and all unmeasured variables (*U*) which confound *X* and *Y*. β is the causal effect of *X* on *Y*. (A) δ is the genetic effect on *X*. Dashed lines and crosses indicate examples of violations of the standard IV assumptions which can lead to bias. (B) Genetic instruments have a direct effect on *Y* (υ), a phenomenon known as horizontal pleiotropy and a violation of IV3. Genetic instruments have a direct effect on *U* (ψ), violation of IV2 and an example of horizontal pleiotropy that violates the InSIDE assumption

The capacity of traditional observational epidemiology to reliably infer whether a health exposure causally influences a disease rests on its ability to appropriately measure and adjust for factors which jointly predict (or confound) the exposure‐outcome relationship. Mendelian randomization (MR)^1^ avoids bias from unmeasured confounding by using genetic variants as instrumental variables (IVs).^2^ For the approach to be valid to test for causality, each specific IV must be robustly associated with the exposure (assumption IV1), independent of any confounders of the exposure and outcome (IV2) and be independent of the outcome given the exposure and the confounders (IV3), as illustrated in Figure 1A.

Two‐sample summary data MR is a design that derives causal effect estimates with summary statistics obtained from two separate samples—one supplying the single nucleotide polymorphism (SNP)‐exposure associations and the other supplying the SNP‐outcome associations^3^, ^4^, ^5^, ^6^—an SNP being the most common type of genetic variation in the genome. If the chosen SNPs are valid IVs, and the causal effect of a unit increase in *X* on the mean value or risk of *Y* is approximately linear in the local region of *X* predicted by these variants^7^ then a simple inverse‐variance weighted (IVW) meta‐analysis of SNP‐specific causal estimates provides an approximately unbiased estimate of this average causal effect. If sufficient heterogeneity exists between the MR estimates across a set of variants, this suggests evidence for violation of one or more of the IV assumptions. This could be due to assumption IV1 being only weakly satisfied by the genetic variants (ie, weak instrument bias).^8^, ^9^ It is however more problematic when the heterogeneity is caused by violations of assumptions IV2 and IV3.^7^, ^10^ The latter violation is commonly known as “horizontal pleiotropy,”^11^ and hereafter referred to as pleiotropy for simplicity. Pleiotropy does not necessarily lead to biased causal effect estimates if it is “balanced,” in the sense that the average pleiotropic bias across SNPs is zero, and the weight each SNP receives in the analysis is also independent of its pleiotropic effect. This latter condition is commonly referred to as the instrument strength independent of direct effect (InSIDE) assumption.^12^, ^13^ However, this assumption is itself unverifiable.

Methods have been developed that are robust to pleiotropy and InSIDE violation. For example, the weighted median estimator^14^ is statistically consistent if 50% of the SNPs are valid IVs (or not pleiotropic). Similarly, mode‐based estimation strategies focus on identifying the largest subset of variants yielding a homogeneous causal estimate, and are consistent when this set is made up of valid IVs.^15^, ^16^ These approaches do not make any assumptions about the nature of the pleiotropy for invalid SNPs—they could violate InSIDE or not. Other approaches, such as MR‐PRESSO^17^ and Radial MR^8^ attempt to detect and remove SNPs that are specifically deemed responsible for bias and heterogeneity in an MR‐analysis, however they assume the remaining SNPs satisfy InSIDE. Finally, the robust adjusted profile score (MR‐RAPS)^9^ uses an adjusted profile likelihood, which penalizes outlying (and hence likely pleiotropic) SNPs using a robust loss function. MR‐RAPS is also naturally robust to weak instrument bias because uncertainty in the SNP‐exposure association estimates is incorporated into its likelihood function.

In this article, we develop a method for pleiotropy robust MR analysis with two‐sample summary data using the general framework of Bayesian model averaging (BMA).^18^ We adapt this general approach to the summary data setting where the SNPs are uncorrelated but potentially pleiotropic. Our approach uses the profile likelihood of MR‐RAPS^9^ as a basis for efficiently modeling the summary data in the presence of weak instrument bias and pleiotropy, but with the addition of an indicator function to denote whether an individual SNP is included or disregarded in the model. We develop a Metropolis‐Hastings BMA algorithm to intelligently search the space models defined by all possible SNP subsets (ie, ≈ $2^{L}$ in the case of *L* SNPs) in order to decide which SNPs to include in the identified set of valid IVs within a given iteration of the Markov chain. The derived posterior distribution is therefore averaged across all selected SNP combinations. We call our method Bayesian set identification Mendelian randomization (BESIDE‐MR). BESIDE‐MR aims to find the largest set of variants that furnish consistent, homogeneous estimates of causal effect, but accounts for model uncertainty, due to the selection of different instrument sets, which we will show is important for preserving the coverage of resulting MR estimates. Our one‐component BESIDE‐MR model is robust to a small proportion of invalid SNPs, but is inadequate when a large proportion of SNPs are invalid. To address this case, we extend BESIDE‐MR to a two‐component model.

In Section 2, we introduce the methodology behind our one‐component model and in Section 3 assess its performance in Monte‐Carlo simulations. In Section 4, we introduce and assess the performance of the two‐component model extension. In Section 5, both the one‐ and two‐component approaches to investigate the causal role of the amount of cholesterol in extra large high density lipoprotein particles on the risk of age related macular degeneration (AMD) using data from the 2019 MR Data Challenge.^19^ We conclude with a discussion and point to further research.

## 2 METHOD

### 2.1 Description of the general model

Suppose that we have data from an MR study consisting of *N* individuals, where for each subject *k* we measure *L* independent genetic variants ($G_{k1}\ldots G_{kL}$), an exposure ($X_{k}$) and an outcome ($Y_{k}$). $U_{k}$ represents the shared residual error between *X* and *Y* due to confounding, which we wish to overcome using IV methods. To estimate the average causal effect, we assume the following linear structural models^20^ for *U*, *X*, and *Y* consistent with Figure 1B:

$$\begin{array}{ll} U_{k}|G_{k} & =\overset{L}{\underset{j=1}{\sum }}\psi_{j}G_{kj}+\epsilon_{k}^{U},\;\\ X_{k}|U_{k},G_{k} & =\overset{L}{\underset{j=1}{\sum }}\delta_{j}G_{kj}+\kappa_{x}U_{k}+\epsilon_{k}^{X},\;\\ Y_{k}|X_{k},U_{k},G_{k} & =\overset{L}{\underset{j=1}{\sum }}\upsilon_{j}G_{kj}+\beta X_{k}+\kappa_{y}U_{k}+\epsilon_{k}^{Y},\; \end{array}$$

where $\epsilon_{k}^{U}$, $\epsilon_{k}^{X}$, and $\epsilon_{k}^{Y}$ are mean zero independent error terms for *U*, *X*, and *Y*, respectively. See Table S1 for a summary of the assumptions required for the estimation of the average causal effect. From these structural models, we can derive the approximate reduced form models for the *G*‐*X* and *G*‐*Y* associations for SNP *j* :

(1) $$\begin{array}{ll} X_{k}|G_{kj} & \approx (\delta_{j}+\kappa_{x}\psi_{j})G_{kj}+\epsilon_{k}^{\prime X}, \end{array}$$

(2) $$\begin{array}{ll} Y_{k}|G_{kj} & \approx [\upsilon_{j}+\kappa_{y}\psi_{j}+\beta (\delta_{j}+\kappa_{x}\psi_{j})]G_{kj}+\epsilon_{k}^{\prime Y}. \end{array}$$

We use “approximate” here because the error terms $\epsilon_{k}^{\prime X}$ and $\epsilon_{k}^{\prime Y}$ are not exactly the same for all *j*—the *j*th residual error term in fact contains common contributions from all other genetic variants not equal to *j*.^7^ This approximation is very accurate in most settings because the genetic variants combined make a very small contribution to the total residual error in each model (eg, typically of the order of 1%‐2%) and the marginal coefficients are estimated from genome‐wide association studies (GWAS) that usually have sample size of hundreds of thousands.^21^ Under this assumption, the following models can then be justified for summary data estimates of the *G*‐*X* (${\hat{\gamma }}_{j}$) and *G*‐*Y* (${\hat{\Gamma }}_{j}$) associations gleaned from fitting (1) and (2):

(3) $${\hat{\gamma }}_{j}∼N(\gamma_{j},\sigma_{Xj}^{2}),\;{\hat{\Gamma }}_{j}|\alpha_{j},\gamma_{j}∼N(\alpha_{j}+\beta \gamma_{j},\sigma_{Yj}^{2}).$$

Here, $\alpha_{j}=\upsilon_{j}+\kappa_{y}\psi_{j}$ and $\gamma_{j}=\delta_{j}+\kappa_{x}\psi_{j}$. Under Model (3), it is assumed that the first study provides ${\hat{\gamma }}_{j}$ and standard errors $\sigma_{Xj}$, and a second study, independent from the first, provides ${\hat{\Gamma }}_{j}$ and standard errors $\sigma_{Yj}$. Both the standard errors are assumed to be fixed and known. As the two studies are independent, we assume that the uncertainty in ${\hat{\gamma }}_{j}$ is independent of the uncertainty in ${\hat{\Gamma }}_{j}$. Model (3) also assumes that SNPs are independent, which can be ensured by performing linkage disequilibrium (LD) clumping in publicly available tools such as PLINK^22^ and MR‐BASE.^23^ The two‐sample design implicitly assumes that SNP *j* associations have identical associations in both studies as they are sampled from the same population. See Supplementary Section A for further justification of the underlying assumptions made to estimate the average causal effect via two‐sample approach.

The individual Wald ratio estimand for SNP *j* from Model (3) is then

$$\beta_{j}=\frac{\Gamma_{j}}{\gamma_{j}}=\beta +\frac{\alpha_{j}}{\gamma_{j}}=\beta +\frac{\upsilon_{j}+\kappa_{y}\psi_{j}}{\delta_{j}+\kappa_{x}\psi_{j}}.$$

From this we see that to reduce the bias of $\beta_{j}$ of SNP *j*, the instrument strength ($\gamma_{j}$) needs to be large, or the pleiotropic effect ($\alpha_{j}$) should be small. Under Model (3), invalid SNPs can be put into two classes:

- InSIDE respecting pleiotropic SNPs for whom $\upsilon_{j}\neq 0$ but $\psi_{j}=0$.
- InSIDE violating pleiotropic SNPs for whom $\upsilon_{j}\neq 0$ and $\psi_{j}\neq 0$.

InSIDE violation occurs in the last case because instrument strength and pleiotropic effects are functionally related due to a shared $\psi_{j}$ component, so that the sample covariance $\hat{\text{Cov}}(\alpha_{j},\gamma_{j})\neq 0$. For the case of InSIDE respecting pleiotropy, we are able to assume the sample covariance is approximately zero for a sufficient number of instruments, since $\upsilon_{j}$ and $\delta_{j}$ are imagined to be themselves generated via independent processes.^7^ In Supplementary Section B, we show, under the simplifying assumption that the SNP‐outcome standard errors are approximately constant and $\kappa_{x}=\kappa_{y}=1$, when ${\hat{\Gamma }}_{j}\to \Gamma_{j}$ and ${\hat{\gamma }}_{j}\to \gamma_{j}$ as N →∞, the approximate bias term for IVW estimator is,

(4) $$\begin{array}{ll} 𝔼[{\hat{\beta }}_{\text{IVW}}]\;\approx \frac{𝔼\left[\sum_{j=1}^{L}{\hat{\Gamma }}_{j}{\hat{\gamma }}_{j}\right]}{𝔼\left[\sum_{j=1}^{L}{\hat{\gamma }}_{j}^{2}\right]}\to \beta +\frac{𝔼\left[\sum_{j=1}^{L}\alpha_{j}\gamma_{j}\right]}{𝔼\left[\sum_{j=1}^{L}\gamma_{j}^{2}\right]}=\beta +\underset{\text{bias term}}{\underbrace{\frac{\hat{\text{Cov}}(\alpha_{j},\gamma_{j})+\bar{\alpha }\;\bar{\gamma }}{\hat{\mathrm{Var}}(\gamma_{j})+{\bar{\gamma }}^{2}}}}. & \end{array}$$

If all SNPs are pleiotropic, but have mean zero ($\bar{\alpha }=0$) and satisfy the InSIDE assumption ($\hat{\text{Cov}}(\alpha_{j},\gamma_{j})=0$), then the standard IVW provides an unbiased estimate of β. MR‐Egger regression is an extension of IVW that can work under the InSIDE assumption even if $\bar{\alpha }\neq$ 0, which is referred to as “directional” pleiotropy. It does this by estimating an intercept parameter in addition to the causal slope parameter. However, its estimates are generally very imprecise and it is not invariant to allele recoding.^24^ Lastly, it cannot separate directional pleiotropy satisfying InSIDE from balanced pleiotropy violating InSIDE, as the intercept reflects the numerator of the bias term, which is a combination of both. This motivates the use of methods that can attempt to detect and down‐weight a small number of variants that may be responsible for either InSIDE violation or directional pleiotropy so that, for the remainder of SNPs left, Model (3) holds approximately with only InSIDE respecting balanced pleiotropy remaining. This is the approach we will initially pursue for BESIDE‐MR, in line with other researchers.^9^, ^17^ Since BESIDE‐MR does not estimate an intercept term, it is therefore invariant to allele recoding, unlike MR‐Egger regression.

### 2.2 BMA over the summary data model

We are interested in searching over the space of all possible models defined by each of the $2^{L}$ subsets in the entire summary data. Let $I=(I_{1},\ldots ,I_{L})$ be the *L*‐length indicator vector denoting whether SNP $G_{j}$ is included ($I_{j}=1$) or not ($I_{j}=0$) in the model. We want to “force” our data to conform to Model (3) with the additional assumption that $\alpha_{j}∼N(0,\tau^{2})$. The parameters of interest are then $\theta =(\beta ,\tau^{2},I$) and with data, *D*, that consists of ${\hat{\gamma }}_{j}$ and ${\hat{\Gamma }}_{j}$, with their standard errors $\sigma_{Xj}$ and $\sigma_{Yj}$, respectively. The joint posterior is

P(θ|D)∝P(D|θ)P(θ),

where P(D|θ) is the likelihood and P(θ) is user specified prior for each of the parameters. We use a random walk Metropolis‐Hastings (M‐H) algorithm for updating the model parameter values, for the specific details see Supplementary Section C. For a given iteration of the Markov chain, the selection of instruments is conditional on the likelihood of the data and the given priors. After the Markov chain has been sufficiently explored, we can obtain posterior distributions for the model parameters and the posterior probability that each individual SNP is valid. This method has been applied within the context of variable selection and model building to reduce bias from many weak instruments^25^, ^26^ and highly correlated instruments.^27^

It has also been previously shown that using a small number of SNPs for two‐sample MR can lead to large violations of the InSIDE assumption by chance (see fig. A.1 in Bowden et al^7^). Small SNP numbers also make estimation of the pleiotropy variance very imprecise. Therefore, we have restricted the M‐H algorithm to explore models that have at least 5 instruments. Given that the BESIDE‐MR model is weak‐instrument robust, it will almost always be possible to include a sufficient number of instruments because it is not necessary to select only “genome‐wide significant” SNPs. This means that it is amenable to a so called “three sample design,” where an external GWAS is used to select SNPs as instruments, before commencing the two sample MR study. Indeed, this is the approach we take in our applied analysis.

#### 2.2.1 The profile score likelihood

For P(D|θ), we use the profile log‐likelihood derived by Zhao et al.^9^ The profile likelihood is particularly well suited to a Bayesian implementation because it enables heterogeneity due to weak instrument bias and pleiotropy to be taken into account, while only having to update three parameters (β, τ and *I*). Generally, a standard Bayesian formulations requires an additional *L* parameters ($\gamma_{1},\ldots ,\gamma_{L}$) to be updated (see, eg, Thompson et al^28^). BESIDE‐MR is therefore not strictly Bayesian, as we have not used the full likelihood.

Specifically we work with likelihood for ($\beta ,\tau^{2}$) given the data $\left(\hat{\gamma },\hat{\Gamma }\right)$ profiled over the parameters $\gamma_{1},\ldots ,\gamma_{L}$. After the incorporation of our indicator vector *I*, the log‐profile likelihood is approximately given by

(5) $$\begin{array}{ll} l(\beta ,\tau^{2},I|\hat{\gamma },\hat{\Gamma }) & \approx -\frac{\sum_{j=1}^{L}I_{j}}{2}\log(2\pi )-\frac{1}{2}\overset{L}{\underset{j=1}{\sum }}I_{j}\left\{\log(\sigma_{Yj}^{2}+\tau^{2})+\left(\frac{{\left({\hat{\Gamma }}_{j}-\beta {\hat{\gamma }}_{j}\right)}^{2}}{\beta^{2}\sigma_{Xj}^{2}+\sigma_{Yj}^{2}+\tau^{2}}\right)\right\}. \end{array}$$

As shown by the derivation in Supplementary Section D, this likelihood allows for heterogeneity due to pleiotropy via $\tau^{2}$, and weak instruments, via $\sigma_{Xj}^{2}$. If we consider that the existing set of instruments have a small $\tau^{2}$, then the likelihood will increase if introducing a new instrument does not lead to a sufficiently large increase the pleiotropy variance, but decrease otherwise. Hence, our BMA algorithm will naturally give more weight to *I*‐vectors that include large set of instruments with homogeneous causal effect estimates. In other words, it tacitly assumes that the true causal effect can be identified by a large set of instruments with a homogeneous MR estimate. This property is reminiscent of the zero modal pleiotropy assumption (ZEMPA)^15^ or the plurality rule that defines the two‐stage hard thresholding (TSHT) approach of Guo et al.^29^ However, the TSHT approach explicitly aims to isolate the largest set of “valid” instruments and base all inference on this single set, which is equivalent to giving a single *I*‐vector a weight of one and all other vectors a weight of zero. BESIDE‐MR is less aggressive, allowing as many distinct *I*‐vectors as are supported by the data to be given weight in the analysis. This feature properly accounts for model uncertainty. Indeed, as subsequent simulations will demonstrate, this yields causal estimates and standard errors that are less prone to under‐coverage than methods which incorporate instrument selection or penalization.

One such method of penalization, also proposed by Zhao et al,^9^ is MR‐RAPS. Instead of being based on likelihood function (5) which uses standard least squares (or $L_{2}$ loss) plus the addition of our indicator function, it uses a robust $L_{1}$ function such as Huber or Tukey loss. This enables the contribution of large outliers to be penalized (ie, reduced) compared to $L_{2}$ loss. Our use of the standard profile likelihood can be viewed as an alternative way to achieving the robustness of MR‐RAPS, by averaging over multiple instruments sets and where more weight is given to homogeneous SNP sets. As MR‐RAPS is a system of nonlinear equations, in some cases the causal effect may not be globally identifiable,^9^ which motivates the use of BMA approach to model average all possible local causal effects. And thus for this reason, convergence is an essential part of BESIDE‐MR implementation to ensure that all plausible models and parameter values have been explored.

BMA implementations tend to favor parsimonious models, that is, models with fewer variables,^18^ therefore, to explore the sensitivity of our BMA procedure to the average number of SNPs included in the model, we include a penalization term within likelihood function (5);

(6) $$\begin{array}{ll} l(\beta ,\tau^{2},I|\hat{\gamma },\hat{\Gamma })+\overset{L}{\underset{J=1}{\sum }}\frac{I_{j}}{2}\eta \text{.} & \end{array}$$

The parameter η dictates the size of models BMA explores the most. Setting a large positive η, the likelihood will increase with number of instruments, then BMA will favor models with many instruments. And hence for negative η, BMA will favor models with fewer instruments. We will assume η to be zero throughout the simulations, but explore ranges of η as sensitivity analysis for the real data example in Section 5.1.

### 2.3 Choice of priors

In general, we encourage the construction of priors to be based on previous epidemiological study or biological knowledge. For the purpose of elucidating our approach, we will use priors that ensure efficient mixing and rapid convergence. For the causal effect parameter β, we use a zero centered normal prior P(β). For the pleiotropy variance ($\tau^{2}$), we use a gamma prior P(Prec) for the precision, where $\text{Prec}=1/\tau^{2}$. For the indicator function prior, we will assume an uninformative Bernoulli prior P(I) with probability $\frac{1}{2}$ for all $I_{j}$. Note a prior probability of 0.5 implies each instrument is equally likely to be pleiotropic or not, and therefore evidence for pleiotropy will be dictated by the likelihood of data. We strongly recommend using biologically informative priors, see Section 6 for further discussion.

### 2.4 An alternative implementation

It is well known that the estimation of $\tau^{2}$ is challenging, even within a classical framework, as its maximum likelihood estimate is not consistent, see Section 4 of Zhao et al^9^ for further discussion. Therefore, we propose an alternative implementation of our M‐H algorithm in which a plug‐in estimate for $\tau^{2}$ is substituted at each iteration. For simplicity, we chose to use the closed‐form DerSimonian‐Laird (DL) estimate for $\tau^{2}$.^30^ In Supplementary Section C, we describe how the M‐H algorithm is modified to implement this alternative approach. Hereafter, we will refer to the first method as the “full Bayesian” approach and this latter method as the DL approach.

## 3 MONTE CARLO SIMULATION

### 3.1 Simulation strategy

We simulate two‐sample summary MR data sets with L=50 instruments from Model (3). Motivated by recent genetic studies,^31^, ^32^ four scenarios are considered;

1. All instruments are strong and invalid instruments have balanced pleiotropy.
2. All instruments are weak and invalid instruments have balanced pleiotropy.
3. All instruments are strong and invalid instruments have directional pleiotropy.
4. All instruments are weak and invalid instruments have directional pleiotropy.

The strength of the instruments is measured by the mean F‐statistic ($\bar{F}$) over all instruments. Pleiotropic effects, $\alpha_{j}$, is simulated from a normal $N(\mu_{\alpha },\sigma_{\alpha })$ distribution, where zero and nonzero $\mu_{\alpha }$ gives balanced and directional pleiotropy respectively, as shown in Table 1. While scenarios 3 and 4 are referred to as directional pleiotropy, it is both indistinguishable and equivalent to the case of InSIDE violating pleiotropy, as illustrated in Equation (4). Within each scenario, 0% to 100% (at 20% intervals) of the *L* SNPs are simulated as invalid instruments. We first compare our approach with the standard IVW method, MR‐APS and MR‐RAPS. The latter two are the classical counterparts that our approach sits between. Specifically, MR‐APS is the MR‐RAPS with a standard $L_{2}$ loss function as opposed to Huber or Tukey loss. We monitor the mean bias of the causal parameter estimate and the coverage (for BESIDE‐MR the bias is taken with respect to the mean of the posterior distribution of β and the coverage is calculated from its credible interval). For BESIDE‐MR only, we also give the posterior probability of inclusion (PPI) in the valid instrument set for each SNP. We also report the weak instrument bias corrected exact *Q*‐statistic^8^ to measure the amount of heterogeneity due to pleiotropy in our simulated data. See details of the simulation strategy in Supplementary Section E.

**TABLE 1:** Summary of simulation scenarios

Scenario	Type of pleiotropy	$\bar{F}$	Pleiotropic effect ($\alpha_{j}$) of invalid instruments
1	Balanced	100	N(0,0.04)
2	Balanced	10	N(0,0.04)
3	Directional	100	N(0.05,0.04)
4	Directional	10	N(0.05,0.04)

From the convergence test (see Supplementary Section E2), our algorithm functions effectively with 50 000 iterations with a burn‐in of 10 000 iterations with the DL and fully Bayesian implementations taking 5 and 7 seconds to converge respectively on a standard desktop computer. Increasing the number of instruments (*L*) increases the potential models ($2^{L}$) for BESIDE‐MR to explore, but for the same number of iterations, convergence was reached even with 100 valid instruments. In rare occasions, we removed results from simulations where the BESIDE‐MR model had failed to converge after 50 000 iterations. For example, for Scenario 1 without invalid instruments, 8 and 7 out of 1000 simulated datasets did not converge for the DL and full Bayesian implementation respectively. This changed to 15 and 29 in the weak instrument case.

### 3.2 Results

Table 2 shows the results. Under Scenario 1, all methods deliver approximately unbiased estimates. The IVW, MR‐APS, and MR‐RAPS estimators achieve nominal coverage when there are no pleiotropic instruments. However, as the proportion of pleiotropic instruments (and hence the heterogeneity) increases, their coverages can drop substantially, with the MR‐APS and MR‐RAPS estimators most affected. BESIDE‐MR has conservative coverage under no heterogeneity (due to many nuisance parameters^33^ in the absence of invalid instruments) but maintains far better coverage when heterogeneity increases. The general pattern remains the same for weaker instruments (Scenario 2), even with many more weak instruments (L=100), with results shown in Supplementary Section E4. In Scenario 3, all the approaches deteriorate with increasing number of invalid instruments, but BMA has consistently the least bias and best coverage throughout. In Scenario 4, the IVW estimator is seemingly least biased, due to weak instrument bias canceling out some of the pleiotropic bias. With 40% and 60% invalid instruments, full Bayesian BESIDE‐MR struggled to converge within 50 000 iterations in a small number of cases.

**TABLE 2:** Evaluation criteria for different types of pleiotropy and instrument strength (Table 1)

		IVW		DL est.		Full Bayes.		MR‐APS		MR‐RAPS	
No. inv.	*Q*	Bias	Cover.	Bias	Cover.	Bias	Cover.	Bias	Cover.	Bias	Cover.
*Scenario 1*											
0	49.0	−0.001	96.40	−0.000	97.50	0.000	98.10	−0.000	94.40	−0.000	94.00
10	57.9	−0.001	93.20	0.000	97.50	0.000	97.70	−0.000	89.50	−0.000	92.10
20	66.4	−0.001	90.80	−0.000	95.40	−0.000	94.60	−0.000	83.90	−0.000	87.30
30	75.5	−0.000	88.30	0.001	94.20	0.001	92.00	0.001	77.30	0.001	80.80
40	84.0	−0.001	86.80	−0.000	95.80	−0.000	90.70	0.001	76.60	0.001	77.60
50	91.9	0.000	85.40	0.000	94.80	0.001	86.60	0.002	70.40	0.001	72.90
*Scenario 2*											
0	48.7	−0.018	33.40	−0.001	97.10	0.002	96.10	−0.000	93.90	−0.000	92.90
10	54.4	−0.019	37.50	−0.000	97.10	0.005	93.70	0.003	91.80	0.003	92.10
20	59.2	−0.018	41.70	0.001	96.70	0.008	90.50	0.006	88.00	0.006	89.10
30	64.0	−0.018	44.60	0.001	96.70	0.011	87.80	0.009	83.20	0.008	84.90
40	68.8	−0.018	46.50	0.001	95.60	0.014	80.20	0.012	72.50	0.011	75.70
50	73.9	−0.019	47.80	0.002	94.60	0.017	73.40	0.015	68.80	0.015	70.10
*Scenario 3*											
0	49.0	−0.001	96.40	−0.000	97.50	0.000	98.10	−0.000	94.40	−0.000	94.00
10	69.0	0.011	75.60	0.007	92.80	0.007	92.70	0.013	61.30	0.009	75.80
20	84.1	0.024	35.20	0.018	71.90	0.016	70.00	0.027	20.20	0.021	33.60
30	92.0	0.037	11.80	0.032	38.20	0.031	36.10	0.039	4.70	0.035	7.90
40	96.1	0.051	1.40	0.049	9.30	0.049	9.70	0.054	0.10	0.052	0.40
50	95.2	0.064	0.30	0.066	1.50	0.067	1.50	0.068	0.00	0.067	0.00
*Scenario 4*											
0	48.7	−0.018	33.40	−0.001	97.10	0.002	96.10	−0.000	93.90	−0.000	92.90
10	58.8	−0.011	69.77	0.007	95.60	0.015	79.00	0.018	66.30	0.016	71.70
20	64.5	−0.003	84.70	0.017	84.60	0.028	46.20	0.035	23.70	0.034	29.60
30	66.5	0.006	82.60	0.028	64.60	0.040	21.70	0.050	5.10	0.048	7.00
40	66.2	0.014	70.10	0.040	35.60	0.049	9.90	0.064	0.40	0.063	0.60
50	65.3	0.022	53.90	0.050	18.90	0.057	5.20	0.075	0.10	0.074	0.10

*Note*: 50 instruments in total. True β is 0.05.

Abbreviations: Bias, mean bias; Cover., coverage; DL est., DL estimate; Full Bayes., full Bayesian; No. inv., number of invalid instrument(s); *Q*, Q‐statistics with exact weights.

**FIGURE 2:**
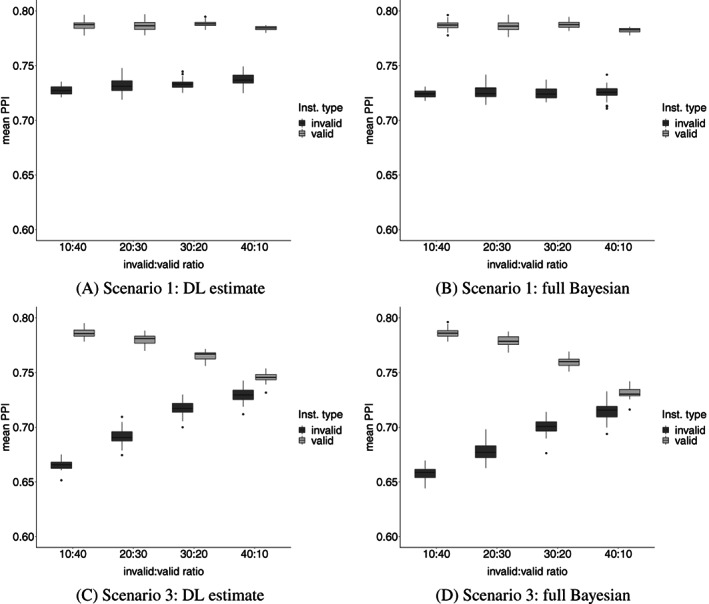
Box plots of PPI for true valid and invalid instruments under balanced and directional pleiotropy (Scenarios 1 and 3, respectively) and under four valid/invalid SNP ratios. (A, C) The DL implementation, and (B, D) the full Bayesian implementation. PPI is posterior probability of inclusion in the valid instrument set

The PPI box plots in Figure 2 demonstrates BESIDE‐MR's ability to distinguish valid from invalid instruments in Scenarios 1 and 3. Under Scenario 1, we see a smaller and constant difference across different proportions of invalid instruments. Under Scenario 3 this difference is maximized (ie, we get the best discrimination) when there are 20% invalid instruments, this difference steadily decreases to half its value as the number of invalid instruments increases further, indicating that BESIDE‐MR generally struggles to deal with directional/InSIDE violating pleiotropy across a substantial proportion of invalid SNPs. There is still a difference in PPI between valid and invalid instruments, however the discrimination is worse for weak instruments. This poor performance with directional/InSIDE violating pleiotropy motivates our two‐component model formulation in Section 4.

Additional simulations were performed to investigate the robustness to non‐normal pleiotropic effect and the effect on PPI with different patterns of heterogeneity. For the former, the difference in bias is minimal between the estimators. The coverage for BESIDE‐MR decreases with increasing number of invalid instruments, but still close to nominal coverage (Supplementary Section E5). For the latter, we find that the discrimination is best with small numbers of highly pleiotropic SNPs, and the worst with large numbers of weakly pleiotropic SNPs. However, the algorithm maintains its reliability even in this case. For further details, see Supplementary Section E6.

## 4 AN EXTENDED TWO‐COMPONENT BMA MODEL FOR INSIDE VIOLATION

The one‐component BESIDE‐MR model introduced thus far assumed that the majority of SNPs were valid under the InSIDE assumption, but a small proportion could be invalid under InSIDE. We now consider the use of an extended model to account for the more extreme case where a large proportion of SNPs may be pleiotropic, and in violation of InSIDE (Figure 1B). In this case, also demonstrated by the previous section, the standard one‐component BESIDE‐MR model cannot easily identify and remove the invalid SNPs, they must instead be formally modeled with an additional slope parameter. To motivate this approach we use the same underlying data generating Model (3). For illustration, suppose that we have two different groups of invalid instruments: in the first group, $S_{1}$, the SNPs exhibit balanced pleiotropy under the InSIDE assumption, but still collectively identify the true causal effect, β. The remaining instruments are in a set $S_{2}$, where the InSIDE assumption is perfectly violated (ie, the correlation between the SNP‐exposure association and the pleiotropic effect is 1). Using the bias formulas in Equation (4), the set of SNPs in $S_{2}$ identify a distinct, biased version of the causal effect (β+1). This data generating model would give rise to two clusters or slopes in the data, which motivates our extended two‐component version of BESIDE‐MR, that is, our model assumes that there are 2 effects to be estimated. In addition, although the main purpose of our extension is to approximate causal effect in the presence of directional/InSIDE violating pleiotropy (these are not distinguishable as shown by Equation 4), this same modeling framework could also account for true mechanistic heterogeneity,^34^ which will be discussed later.

### 4.1 A modified BMA algorithm

Under the data generating Model (3), assume that the pleiotropic effects for InSIDE respecting SNPs in $S_{1}$ are generated from a $N(0,\tau_{1}^{2})$ distribution and InSIDE violating SNPs in $S_{2}$ are from a $N(0,\tau_{2}^{2})$ distribution. They therefore identify a distinct slope parameter. Our total parameter space is modified to $\theta =(\beta_{1},\tau_{1}^{2},\beta_{2},\tau_{2}^{2},I_{1},I_{2})$, with likelihood:

(7) $$\begin{array}{ll} l(\theta |\hat{\gamma },\hat{\Gamma }) & ={\mathrm{Max}}_{\gamma }l(\beta_{1},\tau_{1}^{2},\beta_{2},\tau_{2}^{2}|\hat{\gamma },\hat{\Gamma })\\ & =\log f(\hat{\gamma },\hat{\Gamma }|\beta_{1},\tau_{1}^{2},\beta_{2},\tau_{2}^{2})\\ & \approx -\frac{\sum_{j=1}^{L}I_{1j}}{2}\log(2\pi )-\frac{1}{2}\overset{L}{\underset{j=1}{\sum }}I_{1j}\left\{\log(\sigma_{Yj}^{2}+\tau_{1}^{2})+\left(\frac{{\left({\hat{\Gamma }}_{j}-\beta_{1}{\hat{\gamma }}_{j}\right)}^{2}}{\beta_{1}^{2}\sigma_{Xj}^{2}+\sigma_{Yj}^{2}+\tau_{1}^{2}}\right)\right\}\\ & \;-\frac{\sum_{j=1}^{L}I_{2j}}{2}\log(2\pi )-\frac{1}{2}\overset{L}{\underset{j=1}{\sum }}I_{2j}\left\{\log(\sigma_{Yj}^{2}+\tau_{2}^{2})+\left(\frac{{\left({\hat{\Gamma }}_{j}-\beta_{2}{\hat{\gamma }}_{j}\right)}^{2}}{\beta_{2}^{2}\sigma_{Xj}^{2}+\sigma_{Yj}^{2}+\tau_{2}^{2}}\right)\right\}, \end{array}$$

where the indicator functions $I_{1j}$ and $I_{2j}$ denote whether an SNP *j* is included in $S_{1}$ or $S_{2}$. We impose the condition that $I_{1j}+I_{2j}\leq 1$, which means that, at a given iteration of our BMA algorithm an SNP is either in $S_{1}$ ($I_{1j}=1,I_{2j}=0$), $S_{2}$ ($I_{1j}=0,I_{2j}=1$), or in neither $S_{1}$ or $S_{2}$ ($I_{1j}=I_{2j}=0$), which we give the label $S_{0}$. This gives the model the flexibility to assign an SNP to either $S_{1}$ or $S_{2}$, or remove it from the model completely by assigning it to $S_{0}$. In Supplementary Section F, we give further details on the M‐H algorithm to update the parameter space of this extended model.

The log‐likelihood with the addition of two model complexity penalization terms is then;

(8) $$\begin{array}{ll} l(\theta |\hat{\gamma },\hat{\Gamma })+\overset{L}{\underset{j=1}{\sum }}\frac{I_{1j}}{2}\eta_{1}+\overset{L}{\underset{j=1}{\sum }}\frac{I_{2j}}{2}\eta_{2}. & \end{array}$$

As in Section 2.2.1, we set $\eta_{1}=\eta_{2}=0$ for the simulations, but vary the values as sensitivity in the applied example.

### 4.2 Simulation study

Two‐sample summary data are simulated with 50 SNPs under balanced pleiotropy but with a progressively larger proportion of SNPs maximally violating the InSIDE assumption. This changes the proportion of SNPs that are in set $S_{1}$ and $S_{2}$. These data are simulated under a strong instrument scenario ($\bar{F}=100$, Scenario 5) and a weaker instrument scenario ($\bar{F}=25$, Scenario 6). For precise details of the simulation parameters see Table 3. We also explore the performance of our two‐component model under balanced pleiotropy with weak and strong instruments when there is no InSIDE violation, that is under Scenarios 1 and 2. This means that all SNPs are effectively in set $S_{1}$ and the data can be explained with a single causal slope parameter, rather than two. The full results are shown in Table 4 where we report the bias, coverage and mean *Q*‐statistic with exact weights of all approaches across 1000 simulations, as before. For BESIDE‐MR, ${\text{PPI}}_{S_{1}}$ and ${\text{PPI}}_{S_{2}}$ for each SNP are also reported. This represents the posterior probability of an SNP being included in $S_{1}$ and $S_{2}$ cluster respectively.

**TABLE 3:** Summary of InSIDE simulation scenarios

Scenario	$\bar{F}$ of $S_{1}:S_{2}$	Typeof pleiotropy	$S_{1}$	$S_{2}$
5	100:100	Balanced	$\psi_{j}=0$,	$\psi_{j}∼U(0.34,1.1)$,
			$\upsilon_{j}∼N(0,0.04)$,	$\upsilon_{j}=0$,
			$\delta_{j}∼U(0.34,1.1)$,	$\delta_{j}=0$,
			$\sigma_{Xj}∼U(0.06,0.095)$,	$\sigma_{Xj}∼U(0.06,0.095)$,
			$\beta_{1}=\beta$	$\beta_{2}=\beta +1$
6	25:25	Balanced	$\psi_{j}=0$,	$\psi_{j}∼U(0.34,1.1)$,
			$\upsilon_{j}∼N(0,0.04)$,	$\upsilon_{j}=0$,
			$\delta_{j}∼U(0.34,1.1)$,	$\delta_{j}=0$,
			$\sigma_{Xj}∼U(0.06,0.4)$,	$\sigma_{Xj}∼U(0.06,0.4)$,
			$\beta_{1}=\beta$	$\beta_{2}=\beta +1$

**TABLE 4:** Evaluation criteria for estimating two causal parameters

		Q		Mean bias		Median bias		Coverage	
Est.	Inst. $S_{1}:S_{2}$	$S_{1}$	$S_{2}$	$\beta_{1}$	$\beta_{2}$	$\beta_{1}$	$\beta_{2}$	$\beta_{1}$	$\beta_{2}$
*Scenario 1* ($\beta_{1}=\beta_{2}=\beta$)									
DL est.	50:0	60.2	‐	0.001	0.001	0.001	0.001	100.0	99.8
Full Bayes.	50:0	60.2	‐	0.001	0.001	0.001	0.001	99.7	99.5
*Scenario 5* ($\beta_{1}=\beta$, $\beta_{2}=\beta +1$)									
DL est.	40:10	73.5	10.9	0.007	−0.876	0.001	−0.995	99.4	14.6
	30:20	55.1	23.8	0.003	−0.079	0.001	−0.013	95.9	92.3
	25:25	43.9	30.3	0.005	−0.009	0.001	−0.008	93.9	96.7
	20:30	35.3	36.9	0.053	−0.006	0.004	−0.006	91.0	95.6
	10:40	16.5	49.1	0.906	−0.008	0.988	−0.005	11.1	85.5
Full Bayes.	40:10	73.5	10.9	0.076	−0.287	0.003	−0.027	84.0	69.0
	30:20	55.1	23.8	0.230	−0.218	0.008	−0.009	69.4	76.2
	25:25	43.9	30.3	0.248	−0.182	0.011	−0.008	67.9	79.7
	20:30	35.3	36.9	0.254	−0.122	0.013	−0.002	66.8	86.1
	10:40	16.5	49.1	0.225	−0.041	0.017	0.003	62.4	95.4
*Scenario 2* ($\beta_{1}=\beta_{2}=\beta$)									
DL est.	50:0	58.3	‐	0.002	0.002	0.002	0.002	100.0	100.0
Full Bayes.	50:0	58.3	‐	0.004	0.004	0.004	0.003	99.9	99.9
*Scenario 6* ($\beta_{1}=\beta$, $\beta_{2}=\beta +1$)									
DL est.	40:10	67.6	30.2	0.003	−0.985	0.002	−0.997	99.0	1.4
	30:20	50.3	65.7	0.035	−0.474	0.009	−0.391	97.5	60.1
	25:25	41.3	85.0	0.012	−0.099	0.006	−0.060	94.1	93.3
	20:30	32.8	102.6	0.007	−0.037	0.005	−0.033	94.6	96.8
	10:40	14.8	140.6	0.651	−0.072	0.766	−0.062	41.4	93.8
Full Bayes.	40:10	67.6	30.2	0.001	−0.337	0.003	−0.104	89.8	63.2
	30:20	50.3	65.7	0.022	−0.179	0.008	0.016	84.7	78.5
	25:25	41.3	85.0	0.036	−0.233	0.011	0.013	72.8	80.9
	20:30	32.8	102.6	0.002	−0.332	0.011	0.016	64.3	77.5
	10:40	14.8	140.6	0.364	−1.349	0.987	−0.379	22.3	52.8

*Note*: 50 instruments in total. The true β is 0.05. $S_{1}$ and $S_{2}$ are InSIDE respecting and violating set, respectively.

Abbreviations: DL est., DL estimate; Est., estimator; Full Bayes., Full Bayesian; Inst., instrument(s); *Q*, exact *Q*‐statistics.

### 4.3 Results

For data generated under Scenarios 1 and 2, and so in the complete absence of InSIDE‐violating SNPs in set $S_{2}$, our two slope model correctly identifies β and does not try to estimate a second effect, that is, $\beta_{1}=\beta_{2}$. When the data are generated under the new Scenario 5 we see that, when $S_{1}$ and $S_{2}$ have a similar number of instruments, both $\beta_{1}$ and $\beta_{2}$ can be estimated by the DL implementation of our two‐component model. If the proportion of SNPs in either set is too small, then our algorithm tends to remove them completely and focus on estimating just one slope. The full Bayesian implementation returns mean posterior estimates that are median unbiased but not mean unbiased. This demonstrates a lack of convergence for some of simulated data, and indicates that longer iterations and a more sophisticated procedure for deciding on the M‐H tuning parameter may be required to properly fit the model.

When the data are generated with weaker instruments (Scenario 6), we see a degrading in the performance of all approaches. In particular, see that the effect is worst for $\beta_{2}$. This is because, in our specific simulation, $\beta_{2}$ is larger in magnitude than $\beta_{1}$, which increases both the heterogeneity as measured by the exact *Q* statistic (see Equation 9 in Supplementary Section E1) and the absolute magnitude of weak instrument bias relative to that experienced when estimating $\beta_{1}$. This adversely affected the coverage of the estimates. This heterogeneity is further exaggerated with even weaker instruments ($\bar{F}=10$), leading to our approach not being able to correctly assign instruments to either $S_{1}$ or $S_{2}$ (Supplementary Section F). If this case is encountered in practice, we recommend use of the single slope model instead.

When applying the full Bayesian implementation of BESIDE‐MR in Scenario 6, we noticed an important feature most prominent when there was a large imbalance in the relative sizes of $S_{1}$ and $S_{2}$. In this case, the M‐H algorithm can switch from estimating the posterior for $\beta_{1}$ to estimating the posterior for $\beta_{2}$. This problem is referred to as “label switching.”^35^ In our applied analysis in Section 5, we discuss this issue in more detail, and our proposal for addressing it.

**FIGURE 3:**
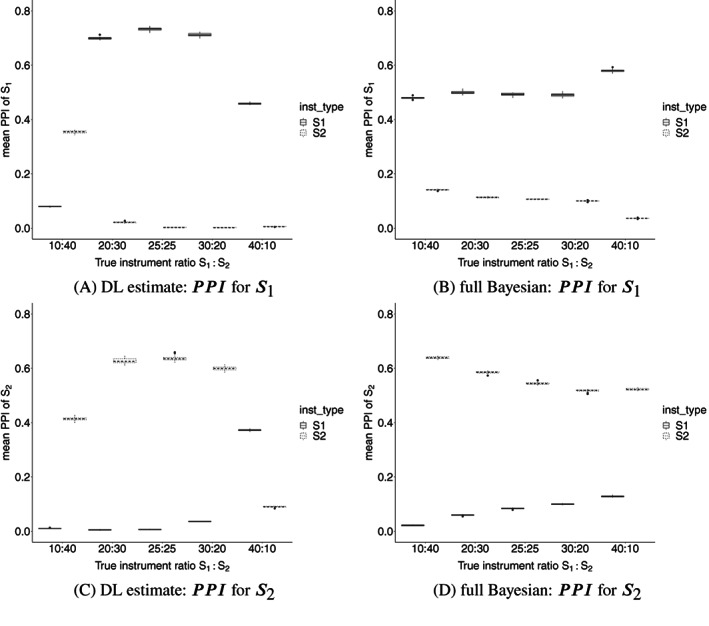
Box plots of ${\text{PPI}}_{S_{1}}$ (A, B) and ${\text{PPI}}_{S_{2}}$ (C, D) of true $S_{1}$ and $S_{2}$ instruments for Scenario 5. *x*‐axis shows the true ratio of instruments in each cluster ($S_{1}$:$S_{2}$), and the *y*‐axis is the average ${\text{PPI}}_{S_{1}}$ and ${\text{PPI}}_{S_{2}}$ of 1000 simulations. (A, C) The DL implementation, and (B, D) the full Bayesian implementation. ${\text{PPI}}_{S_{1}}$ and ${\text{PPI}}_{S_{2}}$ are the posterior probability of an SNP being included in $S_{1}$ and $S_{2}$ cluster respectively

Figure 3 gives further insight into how well the DL and full Bayesian implementations can correctly partition the SNPs into clusters. The *x*‐axis shows the true ratio of SNPs in $S_{1}$ and $S_{2}$ and the *y*‐axis shows mean PPI for true $S_{1}$ and $S_{2}$ SNPs separately. For the DL approach (Figure 3), true $S_{1}$ and $S_{2}$ SNPs are correctly assigned higher PPI for $S_{1}$ and $S_{2}$ cluster respectively when there are approximately equal numbers of SNPs in $S_{1}$ or $S_{2}$ (ie, 25:25, 30:20, or 20:30), but true $S_{2}$ SNPs have the same PPI for the $S_{1}$ and $S_{2}$ clusters when the ratio is more unequal (ie, 10:40 or 40:10). This is because the DL approach more aggressively prefers to estimate one parameter only, and treats minority SNPs as outliers (eg, assign to $S_{0}$). By contrast, PPI for the full Bayesian approach is much more constant across all ratios and is also consistently lower. When the $S_{1}$:$S_{2}$ ratio is balanced, both implementations correctly identified $S_{1}$ and $S_{2}$ instruments. However, as explained above, due to greater magnitude in heterogeneity in estimating $\beta_{2}$, both implementations struggle to identify $S_{2}$ SNPs with weaker instruments (Figures S7 and S8).

If an SNP increases the overall heterogeneity ($\tau^{2}$) in either cluster, BESIDE‐MR increasingly classes it as belonging to $S_{0}$ (neither $S_{1}$ nor $S_{2}$). Using a simulated example, Figure S9 demonstrates that the further the SNP is from either of the slope lines, the higher (darker in color) the probability it belongs to neither cluster.

## 5 APPLIED EXAMPLE: AGE‐RELATED MACULAR DEGENERATION AND CHOLESTEROL

Age‐related macular degeneration (AMD) is a painless eye‐disease that eventually leads to vision loss. Recent GWAS have identified several rare and common variants located in gene regions that are associated with lipid levels,^36^ fuelling speculation as to whether the relationship is causal.^37^, ^38^ To this end, a multivariable MR analysis was performed by Burgess and Davey,^39^ which provided evidence to support a causal relationship between AMD and HDL cholesterol but not with LDL cholesterol and triglycerides. In follow up work, Zuber et al^40^ fitted a multivariable MR model using BMA, with a total of 30 separate lipid fraction metabolites acting as the intermediate exposures. Out of the 30, large particle HDL cholesterol (XL.HDL.C) had the highest inclusion probability as a risk factor for AMD.

Although multivariable MR approaches can remove bias due to pleiotropy via known pleiotropic pathways (in this case, other lipid fractions), they can be much more challenging to fit, especially when the correlation between the included exposures is high. For this reason we now revisit this data and use our univariate MR approaches to probe the causal relationship between XL.HDL.C and AMD.

**FIGURE 4:**
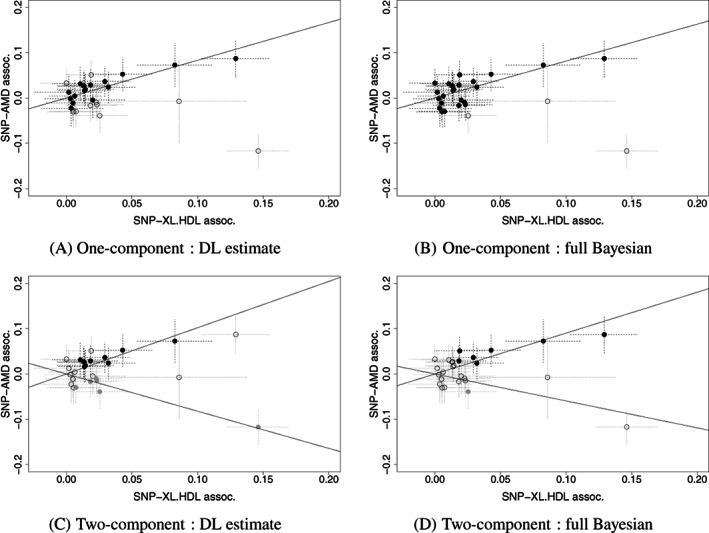
AMD and HDL: Scatter plot of the relationship between SNP‐outcome and SNP‐exposure association, where the filled SNPs had PPI>0.75. (A, B) The one‐component model for DL and full Bayesian approach, respectively, and the solid line is the estimated β. (C, D) Two‐component model for DL and full Bayesian approach respectively, and black and gray colored SNPs are instruments that had strong evidence for cluster $I_{1}$ that estimated $\beta_{1}$ and $I_{2}$ for $\beta_{2}$, respectively. The solid lines are the estimated $\beta_{1}$ and $\beta_{2}$

For our three‐sample MR,^41^ we selected 27 instruments from the METSIM study,^42^ the association of G‐X and G‐Y summary statistics for the chosen instruments are extracted from Kettunen et al^43^ and Fritsche et al.^36^ To avoid severe weak instrument bias,^44^ instruments were chosen based on their individual F‐statistics with XL.HDL.C to be greater than 3, which gives combined mean F‐statistics of 10. A scatter plot for these data is shown in Figure 4. The results for our various data analyses are given in Table 5.

**TABLE 5:** Estimates for the causal effect of a unit increase in XL.HDL.C on the risk of AMD using a range of methods

Parameters	Estimator	Mean	95% Lower Interval	95% Upper Interval
*Standard one‐component approaches*				
β	IVW	0.0251	−0.3493	0.3995
	MR‐APS	0.0672	−0.2997	0.4341
	MR‐RAPS	0.4567	0.1350	0.7785
*BESIDE‐MR: One‐component model*				
β	DL estimate	0.8331	0.5332	1.2679
	Full Bayesian	0.8149	0.5050	1.2105
$\tau^{2}\times 10^{4}$	DL estimate	0.0024	0.0000	0.0000
	Full Bayesian	0.3773	0.0833	1.4330
*BESIDE‐MR: Two‐component model*				
$\beta_{1}$	DL estimate	1.0219	0.6229	1.6596
	Full Bayesian	0.9027	0.4998	1.4966
$\beta_{2}$	DL estimate	−0.8212	−1.2022	−0.4983
	Full Bayesian	−0.5948	−1.2456	1.0716
$\tau_{1}^{2}\times 10^{4}$	DL estimate	0.0033	0.0000	0.0000
	Full Bayesian	0.3435	0.0807	1.2606
$\tau_{2}^{2}\times 10^{4}$	DL estimate	0.0061	0.0000	0.0000
	Full Bayesian	0.3735	0.0823	1.4568

When one‐component causal models are fitted to the data, all methods estimate a positive causal effect, with BESIDE‐MR and IVW giving the largest and smallest effect estimates, respectively. This is not surprising because the IVW estimate is known to be vulnerable to weak instrument bias and is biased toward zero in this setting. Figure 4A,B shows instruments with high probability of inclusion (PPI>0.75), using our two implementations. The DL approach is selecting or de‐selecting instruments more aggressively than the full Bayesian approach.

Next, we fit our two‐component causal model, which offers increased robustness to SNPs violating the InSIDE assumption. Interestingly, we see that this estimates two distinct causal effects of opposite sign (Table 5). For the DL approach, approximately 6 SNPs have evidence for inclusion (PPI>0.75) to each of the 2 clusters, see Figure 4C. For the full Bayesian approach, 4 instruments have evidence of inclusion in the set identifying a positive relationship and only SNP *rs903319* for the negative relationship (hence 0 is within the credible interval for this smaller set), see Figure 4D. Figure S10 shows PPI for each instrument.

The DL approach estimates $\tau^{2}$ to be zero. First‐order weights were used to derive the *Q*‐statistics that form part of the DL estimate for $\tau^{2}$ (shown in Supplementary Equation 9) which could potentially have led to an underestimation of the amount of heterogeneity with weak instruments.^8^

Our tentative conclusion is that a small proportion of InSIDE‐violating SNPs act to reduce the apparent causal effect of XL.HDL.C on AMD detectable by a one‐component model. Once this set has been accounted for within a two‐component model, this increases the evidence in favor of a causal role of XL.HDL.C on AMD further. Our results are consistent with Zuber et al^40^ who also found subsets of SNPs which suggested qualitatively different conclusions about the causal role of XL.HDL.C on AMD.

### 5.1 Sensitivity with penalization for model complexity

**FIGURE 5:**
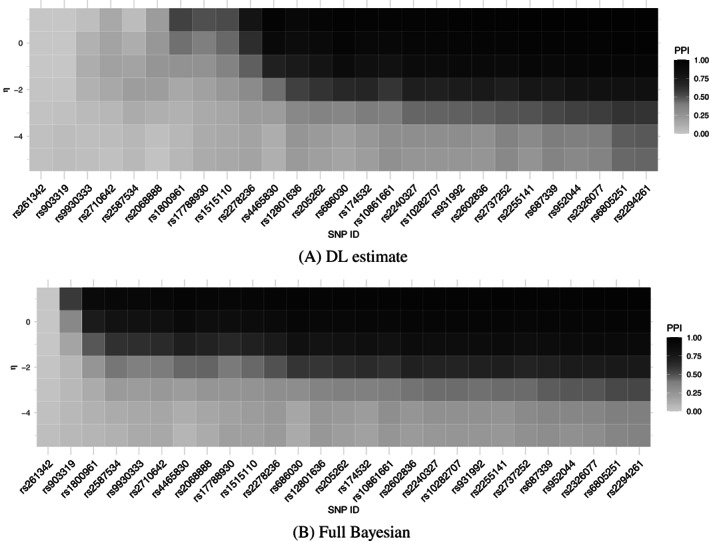
AMD and HDL: Heatmap of η sensitivity on the inclusion probability of each instrument for one‐component model with DL estimate (A) and full Bayesian approach (B)

In the simulations, the penalization parameter for model complexity, η is zero, here we vary η between −5 and 5 for the one‐component BESIDE‐MR. Large negative η would force BESIDE‐MR to favor models with fewer instruments and large positive η would favor many instruments (Tables 6 and S9 for η 2‐5). Furthermore, the heat map of η and PPI in Figure 5 shows that the PPI decreases with η in general, however there are a few instruments that have consistently higher probability for inclusion and *rs261342* is never chosen. The overall causal estimate did not change with η, but with fewer instruments the BESIDE‐MR estimate becomes more uncertain. Similar patterns were found for the two‐component model, see Table S10. The similarity in causal estimates between ranges of η demonstrates that our applied example exhibits large heterogeneity and therefore only a handful of SNPs strongly influencing the results. The inclusion probability is reduced for most instruments, however the inclusion probability for the originally assigned cluster (when $\eta_{1}=\eta_{2}=0$) is still higher than that for the other clusters which further demonstrates the robustness to change in η (Figures S11 and S12).

**TABLE 6:** Sensitivity analysis

η		−5			−4			−3			−2		
Para.	Est.	Med.	LCI	UCI	Med.	LCI	UCI	Med.	LCI	UCI	Med.	LCI	UCI
β	DL	0.78	−1.28	2.02	0.82	−1.21	2.03	0.90	−0.87	1.96	0.91	0.39	1.73
	Bayes	0.70	−1.41	2.02	0.76	−1.29	2.03	0.85	−0.95	1.95	0.87	0.29	1.71
$\tau^{2}\times 10^{-4}$	DL	0.00	0.00	0.00	0.00	0.00	0.00	0.00	0.00	0.00	0.00	0.00	0.00
	Bayes	0.25	0.08	1.43	0.24	0.08	1.41	0.24	0.08	1.29	0.24	0.08	1.23
$\hat{\text{Q}}$	DL	0.56	0.11	0.98	0.61	0.12	0.98	0.75	0.22	0.99	0.87	0.46	1.00
	Bayes	1.02	0.15	2.43	1.04	0.18	2.37	1.12	0.31	2.13	1.28	0.69	1.93
$\sum I_{j}$	DL	5	5	7	6	5	9	8	5	13	13	9	17
	Bayes	5	5	7	6	5	9	9	5	13	15	10	19

η		−1			0			1		
Para.	Est.	Med.	LCI	UCI	Med.	LCI	UCI	Med.	LCI	UCI
β	DL	0.85	0.53	1.49	0.82	0.53	1.26	0.80	0.53	1.12
	Bayes	0.83	0.49	1.43	0.80	0.50	1.21	0.78	0.50	1.12
$\tau^{2}\times 10^{-4}$	DL	0.00	0.00	0.00	0.00	0.00	0.00	0.00	0.00	0.00
	Bayes	0.25	0.08	1.29	0.26	0.08	1.44	0.27	0.09	1.63
$\hat{\text{Q}}$	DL	0.93	0.69	1.00	0.96	0.80	1.00	0.98	0.86	1.00
	Bayes	1.45	1.03	1.95	1.57	1.25	1.95	1.77	1.41	1.94
$\sum I_{j}$	DL	17	13	19	19	16	20	20	18	20
	Bayes	20	15	23	23	20	25	25	22	26

*Note*: Med., LCI and UCI are the median of the posterior distribution with 95% upper and lower credible intervals, respectively. $\hat{\text{Q}}$ is instrument normalized *Q*‐statistics, $\sum Q_{j}/I_{j}$. $\sum I_{j}$ is the number of instruments included. The *Q*‐statistic for 27 instruments is 115.99.

In the simulations, two‐component BESIDE‐MR tends focus on estimating one β when there is an imbalance of instruments in clusters. However, in this sensitivity analysis, BESIDE‐MR consistently estimates two separate slopes over all choices of the model complexity penalization terms. This gives us confidence that the clusters are both real and robustly identified.

### 5.2 Detecting and adjusting for label switching in the full Bayesian model

**FIGURE 6:**
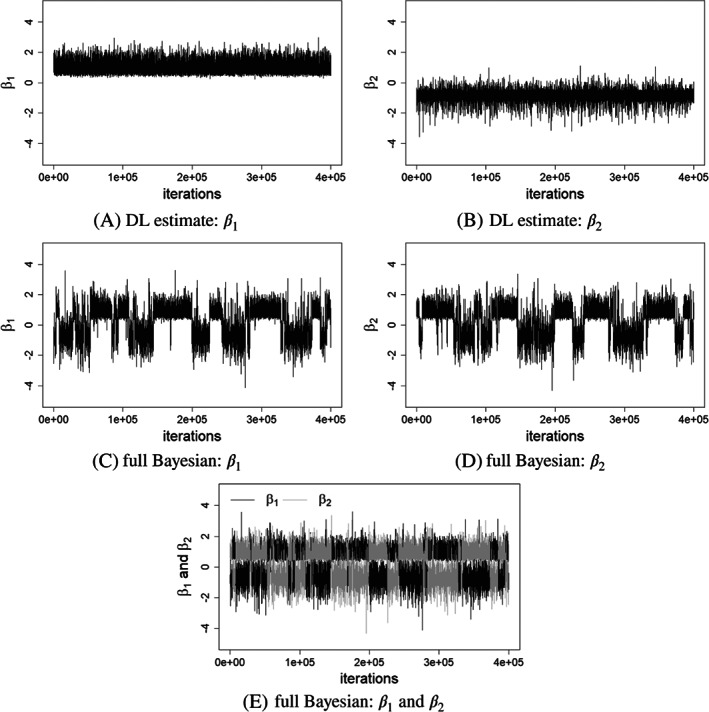
AMD and HDL: Trace plots for $\beta_{1}$ and $\beta_{2}$ from the full Bayesian (A, B) and DL implementations (C, D). And combined $\beta_{1}$ and $\beta_{2}$ for full Bayesian (E)

The trace plots in Figure 6A,B show that the DL implementation consistently identifies two separate distributions for $\beta_{1}$ and $\beta_{2}$, which are centered around 1.02 and −0.82, respectively. This is not the case, however, under the full Bayesian implementation. Trace plots in Figure 6C,D show that the chains for $\beta_{1}$ and $\beta_{2}$ jumping between two distinct values. This is commonly known as “label switching.” One accepted approach for dealing with label switching is to re‐allocate iteration labels from the MCMC output.^35^ We adopted this approach, using a K‐means clustering analysis.^45^ Before K‐means correction, the posterior means of $\beta_{1}$ and $\beta_{2}$ were 0.13 and 0.18, respectively. K‐means analysis clustered 181 186 iterations centered at 0.90 and the second cluster contains 218 815 iterations with mean of −0.59. We re‐assigned the estimates (to $\beta_{1}$ and $\beta_{2}$) accordingly (see Figure 6E) which gave new posterior distribution with mean and credible interval shown in Table 5. This issue further emphasizes the importance of carefully implementing the fully Bayesian approach, and for checking MCMC output for convergence issue. An alternative approach to dealing with label switching is to impose an order restriction on the parameter space, for example so that $\beta_{1}>\beta_{2}$ (we thank a reviewer for this suggestion). However, this lead to poor mixing in the MCMC run which we could not adequately address and we did not pursue this approach further.

## 6 DISCUSSION

In this article, we propose a BMA approach for two‐sample summary data MR that offers robustness to pleiotropy and weak instruments. Our approach can be viewed as a Bayesian extension of the classical MR‐RAPS approach. Rather than assuming, as MR‐RAPS does, the InSIDE violating SNPs are small in number and can be effectively penalized in the analysis, our two‐component formulation allows many invalid SNPs to be incorporated into the analysis to identify a second slope. We were able to demonstrate the potential utility of this extended model in our applied example to uncover sub‐signals in the data that would be missed by conventional methods. We explored two implementations of BESIDE‐MR, namely the full Bayesian and the simplified DL implementation. Our simulations showed that the DL implementation generally performed well, and led to a more aggressive selection of SNPs as either in or out of the model than the full Bayesian approach. It was also much more straightforward to fit, achieve convergence and in our applied analysis did not suffer from label switching. Despite the fully Bayesian implementation requiring more computational time and careful consideration of the MCMC output, it is far better at detecting small effects and consistently identifying outlying instruments. In future work we will attempt to improve the reliability of the full Bayesian approach. Specifically, we plan to create a label switching algorithm^46^ for BESIDE‐MR output and specify a more sophisticated procedure for optimizing the tuning parameter for each model parameter separately. In the meantime, we urge users of the full Bayesian approach to manually adapt the tuning parameters and carefully monitor the mixing and convergence of the MCMC chains, which are the essential aspects of the analysis. We also remind the reader that the number of iterations to reach convergence increases with the number of instruments. As seen in Supplementary Section E2, diagnostic tools such as performing multiple chains with different initial values and trace plots are useful in this regard. For a comprehensive tutorial, see Albert^47^ and Lunn et al.^48^

A useful additional output from our BMA approach compared to classical approaches is the inclusion probability for each SNP. This of course necessitates the specification of a prior probability of inclusion, which we fixed at a constant value of $\frac{1}{2}$. Ideally, one should use informative priors where possible. Indeed, there are multiple sources of external information, for example, epigenetic databases and bioinformatic webtools that could be used to achieve this. For example, a genetic variant that is located in a protein coding gene relevant to the pathway between exposure and outcome of interest can be given a higher inclusion prior probability. Conversely, we might give a much lower inclusion prior probability if the variant is located in a gene that is expressed in multiple tissues. Even though here we are advocating the use priors as a way of incorporating external biological knowledge, most of Bayesian methodology focuses on priors to maximize mixing and speed of convergence.^49^ This is another possible future modification to BESIDE‐MR.

The two‐component model allows BESIDE‐MR to estimate a second slope for an (approximately) equally sized instrument set identifying a homogeneous MR estimate. This second slope could represent InSIDE violation or directional pleiotropy, and was our original motivation. However, it is also an equally valid model to account for “mechanistic heterogeneity.” That is, different SNPs perturb the exposure in distinct ways that gives rise to two true causal effects, known as mechanistic heterogeneity.^34^ This possibility of multiple causal effects is explored in recent work by Iong et al.^34^ In future work we plan to explore the utility of BESIDE‐MR in this alternative setting as well. However, to the best of our knowledge, we do not know any approaches that can differentiate between InSIDE violation, directional pleiotropy, or mechanistic heterogeneity without support of biological knowledge.

Zuber et al^40^ proposed a BMA implementation of multivariable MR,^39^, ^50^ which averages over models incorporating different numbers of exposures rather than instruments. Their approach is able to estimate multiple causal effects but the estimation is subject to weak instruments bias. Our model can in principle be extended to multivariable MR too. For a model with 10 exposure traits, this would necessitate the estimation of 11 causal parameters to account for InSIDE violation via unmeasured pathways. This is a potential topic for future research. A frequentist counterpart of BESIDE‐MR in the individual‐level data setting has been developed by Kang et al.^51^ In summary their first approach takes the union of confidence intervals from models with different combinations instruments that is not rejected by the Sargan test, with user specifying number of invalid instruments as a sensitivity parameter. Their second approach^51^ is a test for evidence against the null hypothesis of no effect which is robust even with only one valid instrument. However, the former approach is computationally intensive for many instruments and for the latter, like all frequentist tests, a *P*‐value cannot distinguish between there being insufficient data to detect an effect or if there is truly no effect. BESIDE‐MR could also be extended to correlated SNPs and 2 dependent samples, both will require additional weights to account for correlation between SNPs for the former, and correlations between the SNP‐exposure and SNP‐outcome association estimates for the latter.

## CONFLICT OF INTEREST

The authors declare no potential conflict of interest.

## Supporting information


**Data S1** Supplementary material

## Data Availability

Software in the form of R code is available on corresponding author's Github (https://github.com/CYShapland/BESIDEMR).
