# Supplementary material for: Profile‐likelihood Bayesian model averaging for two‐sample summary data Mendelian randomization in the presence of horizontal pleiotropy
Source: Stat Med. 2022 Jan 20;41(6):1100–19. doi: 10.1002/sim.9320 (PMC9303808; doi:10.1002/sim.9320)
Supplement: Supplementary file 1 — Data S1 Supplementary material [file SIM-41-1100-s001.pdf]

Supplementary material for: Profile-likelihood Bayesian model averaging for two-sample summary data Mendelian randomization in the presence of horizontal pleiotropy

Chin Yang Shapland[*1,2], Qingyuan Zhao[3], and Jack Bowden[4,1,2]

[1]*MRC Integrative Epidemiology Unit at the University of Bristol, U.K.*
[2]*Population Health Sciences, University of Bristol, U.K.*
[3]*Department of Pure Mathematics and Mathematical Statistics at the University of Cambridge, U.K.*
[4]*College of Medicine and Health at the University of Exeter, U.K.*

[*]Correspondence to chinyang.shapland@bristol.ac.uk

A Assumptions for two-sample MR analyses

Table S1 gives the summary of the assumptions made, which closely follows Table 1 in Bowden *et al.*[1] with exception to NO Measurement Error (NOME) assumption, as the measurement error is computed in our profile likelihood. For the estimation of local average causal effect, additional *structural* assumption is required, that is the model is linear and additive without interactions. The structural assumption could be violated in number of situations [2], in most MR applications scenarios of, binary outcomes and interaction between X and G, is plausible. The former we will discuss in Section 7, and for violation from the latter, approximation of local average causal effect using the linear structural model will still hold in many cases as most of the SNPs effect on X is usually very small [3]. Variation in Instrument Strength (VIS) is reasonable as we assume some sampling error would exist and SNPs used are uncorrelated.

Table S1: Summary of assumptions for two-sample MR analyses. G is the genetic instrument, X is the exposure, Y is the outcome and Z is the unmeasured confounding.

**Assumptions**	**Description**
**Instrumental variable (IV) specific**	
IV1	G is associated with X, not through U ($\gamma_j > 0$).
IV2	G is not associated with U ($\psi_j = 0$).
IV3	No direct effect between G and Y ($\alpha_j = 0$).
Structural	Model (2.1) and (2.2) are linear and without interaction.
**Two-sample Analyses (TSA) specific**	
TSA1	Model (2.1) and (2.2) holds for sample 1 and 2.
TSA2	$\epsilon_k^{\prime Y}$ and $\epsilon_k^{\prime Y}$ in Model (2.1) and (2.2) respectively are independent from each other and other variables.
TSA3	σ^2_{Xj} and σ^2_{Yj} in Model (2.3) are independent and known.
**Instrument Strength Independent of Direct Effect (InSIDE), under IV2**	
General InSIDE	$\widehat{Cov}(\alpha_j, \gamma_j) \to 0$ as $L \to \infty$.
Perfect InSIDE	$\widehat{Cov}(\alpha_j, \gamma_j) = 0$ for analysed sample.
**Variation in Instrument Strength (VIS), under IV2**	
VIS	$\gamma_j \neq \gamma_i$ from G_i and G_j on X.
**One-component BESIDE-MR**	
ZEro Modal Pleiotropy Assumption (ZEMPA)	largest set of instruments with homogeneous causal effect estimates.

B Bias from violation of InSIDE assumption

Suppose we are estimating the causal parameter from instruments that violate the InSIDE assumption using the IVW approach. Its estimand will equal:

$$\hat{\beta}_{IVW} = \frac{\sum_{j=1}^{L} \hat{\Gamma}_j \hat{\gamma}_j}{\sum_{j=1}^{L} \hat{\gamma}_j^2}$$

as the $N \to \infty$, $\hat{\Gamma}_j \to \Gamma_j$ and $\hat{\gamma}_j \to \gamma_j$, so that asymptotically, the expectation of IVW estimate tends towards the following

$$\begin{aligned}
\mathbb{E}[\ \hat{\beta}_{IVW}] \ &\approx \frac{\mathbb{E}\left[\sum_{j=1}^{L} \hat{\Gamma}_j \hat{\gamma}_j\right]}{\mathbb{E}\left[\sum_{j=1}^{L} \hat{\gamma}_j^2\right]} \\
&\to \frac{\widehat{Cov}(\Gamma_j, \gamma_j) + \bar{\Gamma}\bar{\gamma}}{\widehat{Var}(\gamma_j) + \bar{\gamma}^2} \\
&= \frac{\widehat{Cov}(\alpha_j + \beta\gamma_j, \gamma_j) + (\bar{\alpha} + \beta\bar{\gamma})\bar{\gamma}}{\widehat{Var}(\gamma_j) + \bar{\gamma}^2} \\
&= \frac{\widehat{Cov}(\alpha_j, \gamma_j) + \beta\widehat{Var}(\gamma_j) + \bar{\alpha}\bar{\gamma} + \beta\bar{\gamma}^2}{\widehat{Var}(\gamma_j) + \bar{\gamma}^2} \\
&= \beta + \underbrace{\frac{\widehat{Cov}(\alpha_j, \gamma_j) + \bar{\alpha}\bar{\gamma}}{\widehat{Var}(\gamma_j) + \bar{\gamma}^2}}_{\text{bias term}}
\end{aligned}$$

When InSIDE is perfectly violated (α_j=γ_j) the numerator and denominator of the bias term are equal. Therefore, β_{IVW} =β+1

C Metropolis-Hastings algorithm for the one-component causal model

For updating the model parameter values, instead of using the standard Gibbs sampling, where it requires conditional posterior distribution, we used a random walk M-H algorithm to give a proposal distribution for each parameter. Let $\theta_i = (\beta_i, \tau_i^2, I_i)$ be the current ith value of the parameter vector θ. θ_i is updated to θ_{i+1} one parameter at a time, by simulating a candidate value θ^* from proposal density, until it is accepted. Note that if the proposal density $C()$ for a given parameter is 'symmetric' - that is if $C(\theta_i|\theta_{i+1}) = C(\theta_{i+1}|\theta_i)$ then the proposal density can be omitted from the calculation of the acceptance probability. This is the case for β and I, but not τ^2.

C.1 The full Bayesian implementation

- **Update** β

  1. Sample $\beta^* \sim \beta_i + h_\beta N(0,1)$, where h_β is a user defined tuning constant.
  2. Accept $\beta_{i+1} = \beta^*$ with probability:

  $$prob = \min\left\{1, \frac{P(\beta^*, Prec_i, I_i)}{P(\beta_i, Prec_i, I_i)}\right\}$$

  otherwise set $\beta_{i+1} = \beta_i$, where $P(,)$ is the posterior density.

- **Update** $Prec$ ($\tau^2 = 1/Prec$)

  1. Sample

  $$\begin{aligned} &Prec^* \sim U(\mathrm{LB}_{Prec^*}, \mathrm{UB}_{Prec^*}) \\ &\mathrm{LB}_{Prec^*} = \max(\mathrm{LL}, Prec_i - h_{Prec}) \\ &\mathrm{UB}_{Prec^*} = \min(\mathrm{UL}, Prec_i + h_{Prec}) \end{aligned}$$

  where $U(,)$ is the proposal density in the form of a uniform distribution. LL and UL is user defined lower and upper limit for $Prec$ respectively, and h_{Prec} is a user defined tuning constant.

  2. Accept $Prec_{i+1} = Prec^*$ with probability:

  $$prob = \min\left\{1, \frac{U(\mathrm{LB}_{Prec_i}, \mathrm{UB}_{Prec_i})P(\beta_{i+1}, Prec^*, I_i)}{U(\mathrm{LB}_{Prec^*}, \mathrm{UB}_{Prec^*})P(\beta_{i+1}, Prec_i, I_i)}\right\}$$

  Otherwise set $Prec_{i+1} = Prec_i$, where $P(,)$ is the posterior density.

- **Update** I

1. Generate a random number between 1 and L from $P(I_L)$, define it as I_q^*, which is the qth element of I^*.
2. Set $I_d^* = I_{id}$ for all $d \neq q$.
3. Set $I_q^* = (I_{iq} - 1)^2$ (this defines the proposed and current model to differ by one instrument).
4. If $\sum_{j=1}^{L} I_j \geq 5$ is true, continue to the next step, otherwise repeat step 1 (ensures there is enough IVs for estimation).
5. Accept $I_{i+1} = I^*$ with probability:

$$prob = \min\left\{1, \frac{P(\beta_{i+1}, Prec_{i+1}, I^*)}{P(\beta_{i+1}, Prec_{i+1}, I_i)}\right\}$$

   otherwise set $I_{i+1} = I_i$, , where $P(,)$ is the posterior density.

The h_β and h_{Prec} acts as tuning parameters for the acceptance rate. That is, the proportion of iterations that θ^* is accepted as θ_{i+1}. Acceptance rates are recommended to be between 0.25 and 0.45 for the random walk M-H algorithm [4]. We follow this guidance in our implementation of the approach in simulations and applied data settings.

C.2 The DL implementation

$\hat{\tau}^2$ is calculated from DerSimonian-Laird estimate [5] and estimated from every proposed value of β and L;

$$\hat{\tau}^2 = \max(0, (Q - (\sum_{j=1}^{L} I_j - 1))/W) \tag{1}$$

where

$$Q = \sum_{j=1}^{L} I_j w_j (\hat{\beta}_j - \beta_{IVW})^2, \quad \beta_{IVW} = \frac{\sum_{j=1}^{L} I_j w_j \hat{\beta}_j}{\sum_{j=1}^{L} I_j w_j}, \quad W = \sum_{j=1}^{L} I_j w_j - \frac{\sum_{j=1}^{L} I_j w_j^2}{\sum_{j=1}^{L} I_j w_j}$$

and $w_j = 1/Var(\hat{\beta}_j)$ respectively. Note that I_j should not be confused with Higgin's I^2 statistic used to quantify heterogeneity in meta-analysis.

- **Update** β

  1. Sample $\beta^* \sim \beta_i + h_\beta N(0, 1)$, where h_β is a user defined tuning constant.

2. Accept $\beta_{i+1} = \beta^*$ with probability:

$$prob = \min\left\{1, \frac{P(\beta^*, \hat{\tau}_i^2, I_i)}{P(\beta_i, \hat{\tau}_i^2, I_i)}\right\}$$

Otherwise set $\beta_{i+1} = \beta_i$, $P(,)$ is the posterior density.

- **Update** L

1. Generate a random number between 1 and L from $P(I_L)$, define it as I_q^*, which is the qth element of I^*.
2. Set $I_d^* = I_{id}$ for all $d \neq q$.
3. Set $I_q^* = (I_{iq} - 1)^2$.
4. If $\sum_{j=1}^{L} I_j \geq 5$ is true, continue to the next step, otherwise repeat step 1 (ensures there is enough IVs for estimation).
5. Accept $I_{i+1} = I^*$ with probability:

$$prob = \min\left\{1, \frac{P(\beta_{i+1}, \hat{\tau}^{2*}, I^*)}{P(\beta_{i+1}, \hat{\tau}_i^2, I_i)}\right\}$$

where $\hat{\tau}^2$ and $\hat{\tau}^{2*}$ is calculated with I_i and I^* respectively. Otherwise set $I_{i+1} = I_i$, where $P(,)$ is the posterior density.

D Derivation of integrated likelihood

Based on the model shown in Equation (3), and the instruments included and excluded have:

$$\alpha_j \sim N(0, \tau^2) \text{ if } I_j = 1, \tag{2}$$

$$\alpha_j \text{ unrestricted if } I_j = 0. \tag{3}$$

then the likelihood function for summary data of the $G - X$ and $G - Y$ can be given by as:

$$\begin{aligned} L(\beta, \tau^2, \boldsymbol{\gamma}, \boldsymbol{\alpha}_{1-I} | \boldsymbol{I}) = \prod_{j=1}^{L} & \left(\frac{1}{\sqrt{2\pi}\sigma_{Xj}} \mathrm{e}^{-\frac{(\hat{\gamma}_j - \gamma_j)^2}{2\sigma_{Xj}^2}} \frac{1}{\sqrt{2\pi}\sqrt{\sigma_{Yj}^2 + \tau^2}} \mathrm{e}^{-\frac{(\hat{\Gamma}_j - \gamma_j \beta)^2}{2(\sigma_{Yj}^2 + \tau^2)}} \right)^{I_j} \\ & \times \left(\frac{1}{\sqrt{2\pi}\sigma_{Xj}} \mathrm{e}^{-\frac{(\hat{\gamma}_j - \gamma_j)^2}{2\sigma_{Xj}^2}} \frac{1}{\sqrt{2\pi}\sigma_{Yj}} \mathrm{e}^{-\frac{(\hat{\Gamma}_j - \gamma_j \beta - \alpha_j)^2}{2\sigma_{Yj}^2}} \right)^{(1-I_j)} \end{aligned} \tag{4}$$

The integrated likelihood of β and τ^2 is then defined as:

$$\bar{L}(\beta, \tau^2 | \boldsymbol{I}) = \int L(\beta, \tau^2, \boldsymbol{\gamma}, \boldsymbol{\alpha}_{1-I} | \boldsymbol{I}) \, \mathrm{d}\pi(\boldsymbol{\gamma}, \boldsymbol{\alpha})$$

for some distribution on $(\boldsymbol{\gamma}, \boldsymbol{\alpha})$. We can approximate the integrated likelihood by using Laplace method:

$$\int_a^b \mathrm{e}^{Mf(x)} \, \mathrm{d}x \approx \sqrt{\frac{2\pi}{M|f''(\hat{x})|}} \mathrm{e}^{Mf(\hat{x})}, \quad \hat{x} = \underset{x}{\operatorname{argmax}} \, f(x)$$

Let $\boldsymbol{\theta} = (\boldsymbol{\gamma}, \boldsymbol{\alpha}_{1-I})$ and assume it is flat, then

$$\begin{aligned} \bar{L}(\beta, \tau^2 | \boldsymbol{I}) &= \int \mathrm{e}^{l(\beta, \tau^2, \boldsymbol{\theta})} \, \mathrm{d}\pi(\boldsymbol{\gamma}, \boldsymbol{\alpha}) \\ &\approx \int \mathrm{e}^{l(\beta, \tau^2, \hat{\theta}) - 1/2 (\boldsymbol{\theta} - \hat{\boldsymbol{\theta}})^T I_{\boldsymbol{\theta}} (\boldsymbol{\theta} - \hat{\boldsymbol{\theta}})} \, \mathrm{d}\boldsymbol{\theta} \\ &\approx \mathrm{e}^{l(\beta, \tau^2, \hat{\theta})} \frac{2\pi^{\|\boldsymbol{\theta}\|_0 / 2}}{|I_{\boldsymbol{\theta}}|^{1/2}}, \end{aligned} \tag{5}$$

where $\|\boldsymbol{\theta}\|_0 = 2L - \sum_{j=1}^{L} I_j$ and $I_{\boldsymbol{\theta}} = \mathrm{d}iag(I_{\boldsymbol{\theta}1}, \cdots, I_{\boldsymbol{\theta}L})$.

We can profile out $\hat{\theta}$ from $l(\beta, \tau^2, \hat{\theta})$ to give the profile likelihood of (β, τ^2):

$$
\begin{aligned}
l(\beta, \tau^2) = \max_{\boldsymbol{\theta}} l(\beta, \tau^2, \hat{\theta}) = & -\frac{\sum_{j=1}^{L} I_j}{2} \log(2\pi) \\
& -\frac{1}{2} \sum_{j=1}^{L} I_j \left\{ \log(\sigma_{Yj}^2 + \tau^2) + \left(\frac{(\hat{\Gamma}_j - \beta \hat{\gamma}_j)^2}{\beta^2 \sigma_{Xj}^2 + \sigma_{Yj}^2 + \tau^2} \right) \right\} \\
& -\frac{\sum_{j=1}^{L} (1 - I_j)}{2} \log(2\pi) \\
& -\frac{1}{2} \sum_{j=1}^{L} (1 - I_j) \log(\sigma_{Yj}^2)
\end{aligned}
$$

Then our integrated likelihood is:

$$2\bar{L}(\beta, \tau^2 | \boldsymbol{I}) = 2l(\beta, \tau^2) + \|\boldsymbol{\theta}\|_0 \log(2\pi) - \log |I_{\boldsymbol{\theta}}|. \tag{6}$$

$I_{\boldsymbol{\theta}}$ is the Fisher information matrix for

$$\boldsymbol{\theta} = \begin{cases} \gamma_j & \text{if } I_j = 1, \\ (\gamma_j, \alpha_j) & \text{if } I_j = 0 \end{cases}$$

so that

$$
\begin{aligned}
I_{\boldsymbol{\theta}j} &= \begin{cases} -E \left(\dfrac{\partial^2 \log L}{\partial \gamma_j^2} \right) & \text{if } I_j = 1, \\ \\ -E \begin{pmatrix} \dfrac{\partial^2 \log L}{\partial \gamma_j^2} & \dfrac{\partial^2 \log L}{\partial \gamma_j \, \partial \alpha_j} \\ \dfrac{\partial^2 \log L}{\partial \alpha_j \, \partial \gamma_j} & \dfrac{\partial^2 \log L}{\partial \alpha_j^2} \end{pmatrix} & \text{if } I_j = 0 \end{cases} \\
&= \begin{cases} \dfrac{1}{\sigma_{Xj}^2} + \dfrac{\beta^2}{\sigma_{Yj}^2 + \tau^2} & \text{if } I_j = 1, \\ \\ \begin{pmatrix} \dfrac{1}{\sigma_{Xj}^2} + \dfrac{\beta^2}{\sigma_{Yj}^2} & \dfrac{\beta}{\sigma_{Yj}^2} \\ \dfrac{\beta}{\sigma_{Yj}^2} & \dfrac{1}{\sigma_{Yj}^2} \end{pmatrix} & \text{if } I_j = 0 \end{cases}
\end{aligned}
$$

therefore the sum of the log determinant of the information matrix is:

$$log|I_{\boldsymbol{\theta}}| = \sum_{j=1}^{L} I_j \log\left(\frac{1}{\sigma_{Xj}^2} + \frac{\beta^2}{\sigma_{Yj}^2 + \tau^2}\right) + (1 - I_j) \log\left(\frac{1}{\sigma_{Xj}^2} \frac{1}{\sigma_{Yj}^2}\right) \tag{7}$$

if $\beta \approx 0$, then

$$
\begin{aligned}
log|I_{\boldsymbol{\theta}}| &\approx \sum_{j=1}^{L} I_j \log(\frac{1}{\sigma_{Xj}^2}) + (1 - I_j) \log(\frac{1}{\sigma_{Xj}^2} \frac{1}{\sigma_{Yj}^2}) \\
&= \sum_{j=1}^{L} (1 - I_j) \log(\frac{1}{\sigma_{Yj}^2}).
\end{aligned}
$$

With this, Equation 6 approximates to,

$$
\begin{aligned}
2\bar{L}(\beta, \tau^2 | \boldsymbol{I}) &\approx 2l(\beta, \tau^2) + (2L - \sum_{j=1}^{L} I_j) \log(2\pi) + \sum_{j=1}^{L} (1 - I_j) \log(\sigma_{Yj}^2) \\
&= -\sum_{j=1}^{L} I_j \log(2\pi) - \sum_{j=1}^{L} I_j \left\{ \log(\sigma_{Yj}^2 + \tau^2) + \left(\frac{(\hat{\Gamma}_j - \beta\hat{\gamma}_j)^2}{\beta^2 \sigma_{Xj}^2 + \sigma_{Yj}^2 + \tau^2} \right) \right\} \\
&- \sum_{j=1}^{L} (1 - I_j) \log(2\pi) + (2L - \sum_{j=1}^{L} I_j) \log(2\pi) \\
&= \sum_{j=1}^{L} (1 - I_j) \log(2\pi) - \sum_{j=1}^{L} I_j \left\{ \log(\sigma_{Yj}^2 + \tau^2) + \left(\frac{(\hat{\Gamma}_j - \beta\hat{\gamma}_j)^2}{\beta^2 \sigma_{Xj}^2 + \sigma_{Yj}^2 + \tau^2} \right) \right\}
\end{aligned}
$$

E Simulations under the one-component model

This section is specifically for one-component BESIDE-MR, that covers Monte Carlo simulation method, and results for convergence, weaker instruments (L=50 and mean F-statistics of 10), many weak instruments (L=100, mean F-statistics of 5 and 10) and sensitivity to strengths of heterogeneity (varied Q-statistics).

E.1 Simulation Method

We simulate two-sample summary MR data sets with L=50 instruments from Model (3) in the main manuscript, where the parameters γ_j were generated from a Uniform $U(0.34, 1.1)$ distribution, σ_{Xj} was generated from a Uniform $U(0.06, UB)$ and σ_{Yj} was generated from a Uniform $U(0.015, 0.11)$ distribution. The upper bound on the G-X association standard error UB was used to determine mean instrument strength - with $0.095 \leq UB \leq 1$ giving mean F-statistics between 10 and 100 respectively. In this setting, the F-statistic for a single SNP can be approximated as $\hat{\gamma}_j^2/\sigma_{Xj}^2$. We defined invalid instruments as SNPs that have non-zero α_j, as there is a direct effect from SNP to outcome, i.e. violation to IV3.

α_j for invalid instruments is simulated from normal $N(\mu_\alpha, 0.04)$ distribution, with the parameter μ_α being used to determine the mean bias induced by including the invalid instruments in the model. The task of BESIDE-MR in the presence of a non-zero μ_α is to give large weight to models which include SNPs for which $\mu_\alpha \approx 0$. As summarised by Table 1 in the main manuscript, $\mu_\alpha = 0$ for the instruments that have balanced pleiotropic effect, and $\mu_\alpha = 0.05$ for directional pleiotropic effect. Apart from a potential non-zero mean bias, the simulated pleiotropic effects satisfy the InSIDE assumption.

For evaluation criteria, we monitor the following quantities across our simulations:

- Mean bias of the causal parameter estimate. For BESIDE-MR we use the mean of the posterior distribution of β to assess this;
- Coverage: For IVW, MR-APS and MR-RAPS this is based on 95% symmetric confidence intervals assuming normality. For BESIDE-MR this is based on a 95% credibility interval;
- The posterior probability of inclusion (PPI) for valid and invalid SNPs set (BESIDE-MR only)

We also report the exact Q-statistic [6]:

$$Q = \sum_{j=1}^{L} w_j(\beta)(\hat{\beta}_j - \beta)^2 \tag{8}$$

where $w_j = 1/Var(\hat{\beta}_j)$. Note that only invalid SNPs which have a non-zero pleiotropic effect make a non-nominal contribution, so that, for a fixed set of pleiotropy parameters $\alpha_1, \ldots, \alpha_L$:

$$E[Q] = \sum_{\alpha_j \neq 0} \frac{\alpha_j^2}{\beta^2 \sigma_{Xj}^2 + \sigma_{Yj}^2} + (L-1) \tag{9}$$

from knowing that [6];

$$Var(\beta_j) = \frac{\beta^2 \sigma_{xj}^2 + \sigma_{yj}^2}{\hat{\gamma}_j^2} \quad \text{and} \quad \hat{\beta}_j = \beta + \frac{\alpha_j + \epsilon_j}{\gamma_j}.$$

E.2 Convergence

Convergence is an important aspect to Bayesian analysis when implemented using MCMC methods, as it is an iterative process, different possible values are explored at each iteration. To investigate convergence, we run 5 short chains, each with random starting values, 50,000 iterations and 10,000 burn-ins. We also run one long chain with 500,000 iterations and 100,000 burn-ins.

We tested convergence on 4 different types of instruments; (1) Scenario 1 and (2) Scenario 2 without invalid instruments, (3) Scenario 1 with 30% invalid instruments (4) Scenario 1 with 100 valid instruments.

Table S2 demonstrates evidence for convergence with 50,000 iterations and 10,000 burn in. The mean, standard deviation and 95% credible interval of the posterior distribution for β are similar between long and shorts chains, in all 4 scenarios. The difference shown between long and short chains are the standard error and the time-series standard error (adjusted for auto-correlation). This is expected as the accuracy for the posterior mean of β increases with number of iterations. The trace plot is another diagnostic tool; it is a continuous line that shows the values a parameter has against the iteration number. A "catepillar" shaped trace plot, and similarities between long and short chains, supports evidence for convergence (Figure S1 and S2). Table S3 gives the PPI of the 10 SNPs from long and short chain. These 10 SNPs were selected because they had the highest PPI in the long chain. The similarity in inclusion probability between the short and long chains for all the 10 instruments and across scenarios (Table S3) demonstrates evidence for convergence in PPI.

Table S2: Convergence diagnostic of Scenario 1 and 2 without invalid, with 30% invalid and Scenario 1 with many instruments by comparing a long and 5 short chains. Each short chain have 50,000 iterations with 5,000 burn-ins and the long chain have 500,000 iterations and 100,000 burn-ins. True β is 0.05. SD, standard deviation; SE, standard error; CI, credible interval; inst., instrument(s).

**Inst. scenario**	**chain**	**mean β**	**SD**	**SE**	**Time-series SE**	**Lower 95% CI**	**Upper 95% CI**
	**DL estimate**						
	1	0.0485	0.0080	0.00004	0.00014	0.0332	0.0640
	2	0.0488	0.0081	0.00004	0.00014	0.0330	0.0643
	3	0.0485	0.0079	0.00004	0.00014	0.0333	0.0643
	4	0.0484	0.0081	0.00004	0.00014	0.0324	0.0645
	5	0.0485	0.0081	0.00004	0.00014	0.0325	0.0642
Strong	Long	0.0485	0.0081	0.00001	0.00005	0.0327	0.0645
and valid	**Full Bayesian**						
	1	0.0486	0.0082	0.00004	0.00014	0.0323	0.0647
	2	0.0489	0.0080	0.00004	0.00013	0.0331	0.0646
	3	0.0487	0.0082	0.00004	0.00014	0.0324	0.0647
	4	0.0487	0.0083	0.00004	0.00013	0.0327	0.0650
	5	0.0491	0.0083	0.00004	0.00015	0.0325	0.0653
	Long	0.0488	0.0083	0.00001	0.00005	0.0326	0.0652
	**DL estimate**						
	1	0.0485	0.0083	0.00004	0.00016	0.0328	0.0652
	2	0.0482	0.0083	0.00004	0.00015	0.0323	0.0648
	3	0.0480	0.0083	0.00004	0.00015	0.0324	0.0649
	4	0.0484	0.0083	0.00004	0.00014	0.0329	0.0652
	5	0.0484	0.0083	0.00004	0.00016	0.0329	0.0648
Weak	Long	0.0482	0.0083	0.00001	0.00005	0.0323	0.0650
and valid	**Full Bayesian**						
	1	0.0483	0.0084	0.00004	0.00014	0.0319	0.0653
	2	0.0481	0.0085	0.00004	0.00015	0.0320	0.0656
	3	0.0485	0.0083	0.00004	0.00015	0.0324	0.0652
	4	0.0485	0.0084	0.00004	0.00016	0.0330	0.0657
	5	0.0480	0.0086	0.00004	0.00015	0.0318	0.0655
	Long	0.0484	0.0085	0.00001	0.00005	0.0322	0.0658
	**DL estimate**						
	1	0.0578	0.0094	0.00005	0.00020	0.0397	0.0768
	2	0.0580	0.0097	0.00005	0.00024	0.0396	0.0776
	3	0.0575	0.0094	0.00005	0.00020	0.0389	0.0767
	4	0.0573	0.0094	0.00005	0.00024	0.0395	0.0767
Strong	5	0.0584	0.0098	0.00005	0.00026	0.0401	0.0789
with	Long	0.0576	0.0095	0.00002	0.00008	0.0391	0.0766
30%							
invalid							

Continued on next page

Table S2 – *Continued from previous page*

Inst. scenario	chain	mean β	SD	SE	Time-series SE	Lower 95% CI	Upper 95% CI
	**Full Bayesian**						
	1	0.0574	0.0097	0.00005	0.00023	0.0384	0.0768
	2	0.0580	0.0096	0.00005	0.00022	0.0394	0.0769
	3	0.0574	0.0097	0.00005	0.00021	0.0385	0.0767
	4	0.0576	0.0096	0.00005	0.00023	0.0394	0.0768
	5	0.0574	0.0096	0.00005	0.00021	0.0384	0.0765
	Long	0.0576	0.0095	0.00001	0.00007	0.0392	0.0764
	**DL estimate**						
	1	0.0498	0.0067	0.00003	0.00015	0.0368	0.0630
	2	0.0499	0.0070	0.00003	0.00017	0.0363	0.0637
	3	0.0499	0.0070	0.00003	0.00017	0.0362	0.0638
	4	0.0499	0.0069	0.00003	0.00016	0.0362	0.0636
	5	0.0498	0.0068	0.00003	0.00016	0.0367	0.0632
Many	Long	0.0500	0.0068	0.00001	0.00005	0.0365	0.0634
and valid	**Full Bayesian**						
	1	0.0500	0.0070	0.00003	0.00016	0.0363	0.0638
	2	0.0497	0.0072	0.00004	0.00017	0.0357	0.0639
	3	0.0503	0.0071	0.00004	0.00017	0.0368	0.0645
	4	0.0501	0.0071	0.00004	0.00018	0.0361	0.0642
	5	0.0499	0.0071	0.00004	0.00017	0.0358	0.0639
	Long	0.0500	0.0070	0.00001	0.00005	0.0363	0.0637

Table S3: PPI of the 10 SNPs from short and long chains to diagnose the convergence of instrument probability. Note that the SNPs shown are the ones with the highest PPI in the long chain and for each scenario these SNPs differs. Each short chain have 50,000 iterations with 5,000 burn-ins and the long chain have 500,000 iterations and 100,000 burn-ins. inst., instrument(s).

Inst. scenario	chain	SNP1	SNP2	SNP3	SNP4	SNP5	SNP6	SNP7	SNP8	SNP9	SNP10
	**DL estimate**										
	1	0.96	0.97	0.95	0.93	0.91	0.92	0.93	0.90	0.91	0.92
	2	0.98	0.96	0.93	0.92	0.92	0.92	0.93	0.93	0.91	0.93
	3	0.97	0.96	0.94	0.94	0.92	0.92	0.92	0.93	0.91	0.91
	4	0.97	0.95	0.92	0.92	0.92	0.91	0.93	0.92	0.92	0.90
	5	0.97	0.95	0.94	0.94	0.93	0.91	0.94	0.92	0.91	0.88
Strong	Long	0.96	0.96	0.94	0.93	0.92	0.91	0.92	0.92	0.92	0.92
and valid	**Full Bayesian**										
	1	0.96	0.96	0.93	0.92	0.92	0.92	0.93	0.91	0.93	0.91

Continued on next page

Table S3 – *Continued from previous page*

Inst. scenario	chain	SNP1	SNP2	SNP3	SNP4	SNP5	SNP6	SNP7	SNP8	SNP9	SNP10
	2	0.95	0.96	0.94	0.94	0.91	0.91	0.91	0.93	0.92	0.91
	3	0.97	0.95	0.93	0.94	0.93	0.92	0.92	0.93	0.90	0.91
	4	0.94	0.96	0.95	0.91	0.91	0.90	0.90	0.88	0.93	0.92
	5	0.96	0.95	0.94	0.91	0.93	0.92	0.94	0.92	0.91	0.91
	Long	0.96	0.96	0.94	0.93	0.92	0.92	0.92	0.92	0.92	0.91
Weak and valid	**DL estimate**										
	1	0.96	0.95	0.94	0.92	0.90	0.91	0.92	0.90	0.90	0.89
	2	0.94	0.96	0.96	0.93	0.92	0.92	0.92	0.91	0.93	0.90
	3	0.96	0.97	0.94	0.95	0.92	0.93	0.91	0.92	0.92	0.91
	4	0.96	0.97	0.92	0.93	0.92	0.92	0.92	0.92	0.91	0.90
	5	0.95	0.96	0.94	0.95	0.93	0.91	0.92	0.90	0.92	0.93
	Long	0.96	0.95	0.94	0.94	0.91	0.92	0.91	0.92	0.92	0.91
	**Full Bayesian**										
	1	0.96	0.97	0.95	0.92	0.90	0.91	0.90	0.91	0.90	0.93
	2	0.94	0.97	0.94	0.93	0.92	0.92	0.92	0.93	0.90	0.93
	3	0.96	0.96	0.94	0.95	0.91	0.89	0.92	0.90	0.92	0.92
	4	0.95	0.97	0.94	0.93	0.90	0.90	0.90	0.91	0.90	0.88
	5	0.95	0.96	0.94	0.94	0.94	0.92	0.92	0.95	0.91	0.93
	Long	0.96	0.96	0.94	0.93	0.92	0.92	0.92	0.91	0.91	0.91
Strong with 30% invalid	**DL estimate**										
	1	0.96	0.97	0.95	0.92	0.94	0.93	0.92	0.88	0.90	0.88
	2	0.94	0.96	0.93	0.93	0.93	0.93	0.93	0.91	0.90	0.91
	3	0.97	0.96	0.93	0.92	0.96	0.93	0.94	0.92	0.93	0.90
	4	0.94	0.95	0.93	0.94	0.94	0.94	0.93	0.91	0.93	0.90
	5	0.96	0.96	0.93	0.93	0.94	0.94	0.95	0.89	0.91	0.89
	Long	0.95	0.95	0.94	0.94	0.93	0.93	0.93	0.92	0.92	0.90
	**Full Bayesian**										
	1	0.94	0.94	0.94	0.94	0.94	0.93	0.95	0.92	0.93	0.90
	2	0.94	0.95	0.93	0.92	0.95	0.95	0.92	0.93	0.87	0.91
	3	0.95	0.95	0.93	0.94	0.95	0.92	0.93	0.91	0.93	0.90
	4	0.97	0.94	0.94	0.93	0.92	0.93	0.90	0.90	0.89	0.91
	5	0.94	0.96	0.94	0.96	0.94	0.94	0.91	0.92	0.92	0.88
	Long	0.96	0.96	0.93	0.93	0.93	0.93	0.93	0.92	0.91	0.90
Many and valid	**DL estimate**										
	1	0.93	0.95	0.96	0.92	0.96	0.96	0.95	0.93	0.91	0.92
	2	0.97	0.98	0.96	0.96	0.94	0.91	0.94	0.94	0.95	0.94
	3	0.96	0.97	0.94	0.94	0.92	0.95	0.94	0.93	0.94	0.90
	4	0.96	0.96	0.96	0.93	0.95	0.94	0.95	0.88	0.97	0.93
	5	0.94	0.95	0.97	0.96	0.94	0.93	0.96	0.93	0.92	0.91
	Long	0.96	0.95	0.96	0.94	0.94	0.94	0.94	0.93	0.94	0.94

Continued on next page

Table S3 – *Continued from previous page*

**Inst. scenario**	**chain**	**SNP1**	**SNP2**	**SNP3**	**SNP4**	**SNP5**	**SNP6**	**SNP7**	**SNP8**	**SNP9**	**SNP10**
	**Full Bayesian**										
	1	0.96	0.95	0.95	0.95	0.96	0.94	0.95	0.91	0.93	0.94
	2	0.96	0.95	0.97	0.95	0.93	0.93	0.94	0.95	0.95	0.94
	3	0.94	0.97	0.97	0.96	0.94	0.94	0.90	0.92	0.95	0.95
	4	0.96	0.95	0.94	0.93	0.96	0.94	0.94	0.92	0.93	0.95
	5	0.97	0.97	0.94	0.94	0.92	0.92	0.95	0.92	0.88	0.94
	Long	0.95	0.95	0.95	0.95	0.94	0.94	0.94	0.94	0.94	0.94

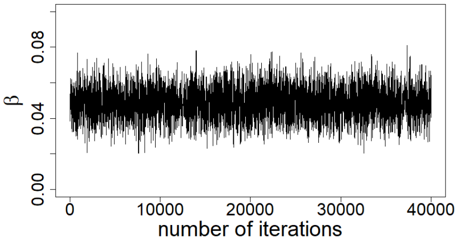

(a) Strong and valid: short

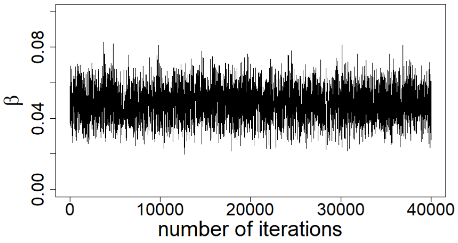

(b) Strong and valid: long

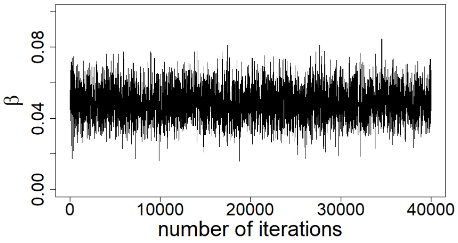

(c) Weak and valid: short

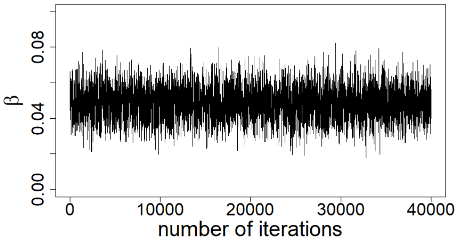

(d) Weak and valid: long

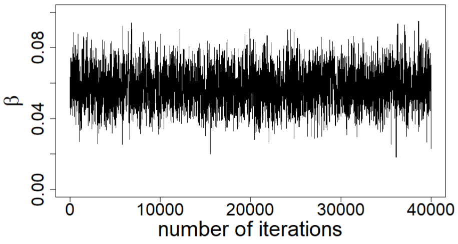

(e) Strong with 30% invalid: short

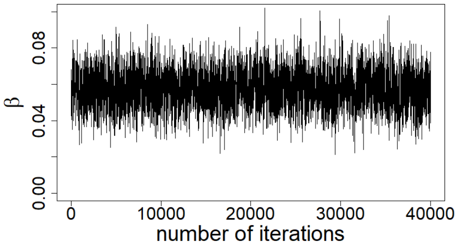

(f) Strong with 30% invalid: long

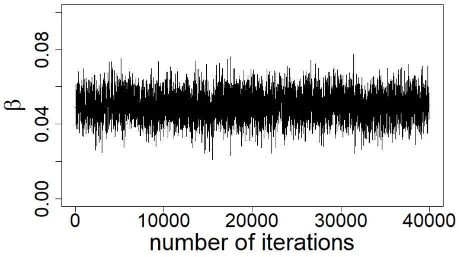

(g) Many and valid: short

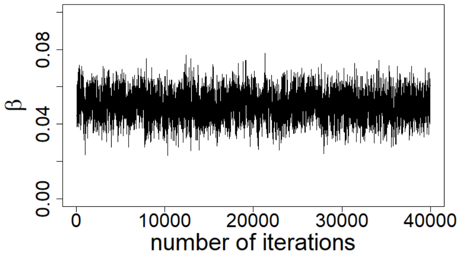

(h) Many and valid: long

Figure S1: Trace plot of the causal effect estimate (β) from DL approach with 3 different instrument scenarios; (a, b) strong valid, (c, d) weak valid instruments only, (e, f) strong with 30% invalid instruments and (g, h) many strong valid instruments. Short and long chain consist of 50,000 and 500,000 iterations with 10,000 and 100,000 burn-in respectively.

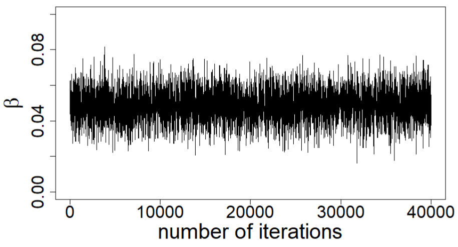

(a) Strong and valid: short

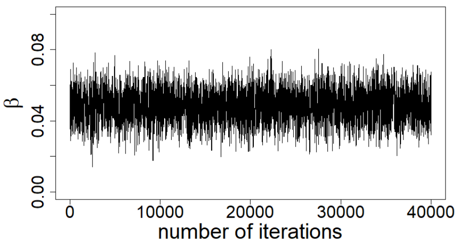

(b) Strong and valid: long

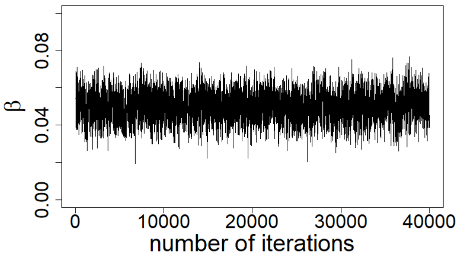

(c) Weak and valid: short

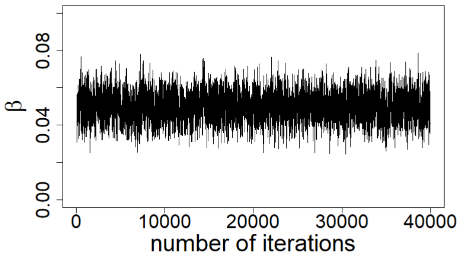

(d) Weak and valid: long

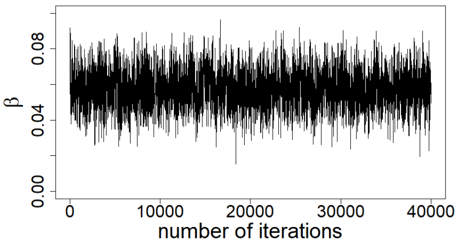

(e) Strong with 30% invalid: short

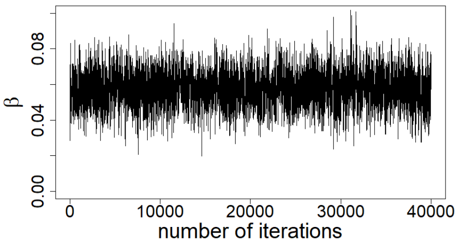

(f) Strong with 30% invalid: long

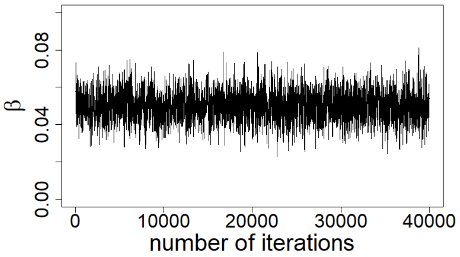

(g) Many and valid: short

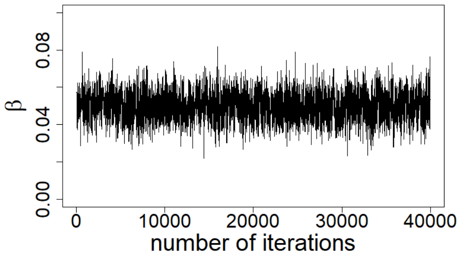

(h) Many and valid: long

Figure S2: Trace plot of the causal effect estimate (β) from full Bayesian approach with 3 different instrument scenarios; (a, b) strong valid, (c, d) weak valid instruments only, (e, f) strong with 30% invalid instruments and (g, h) many strong valid instruments. Short and long chain consist of 50,000 and 500,000 iterations with 10,000 and 100,000 burn-in respectively.

E.3 Weaker instruments

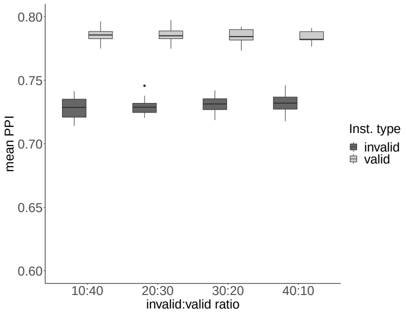

(a) DL estimate

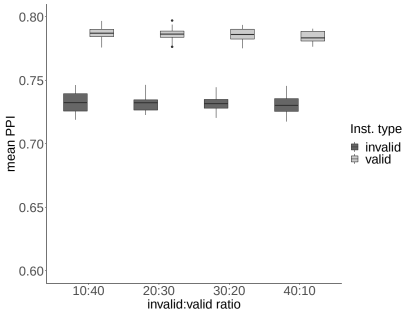

(b) full Bayesian

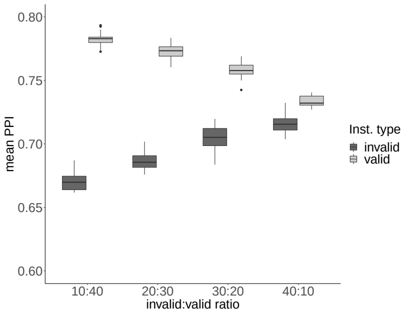

(c) DL estimate

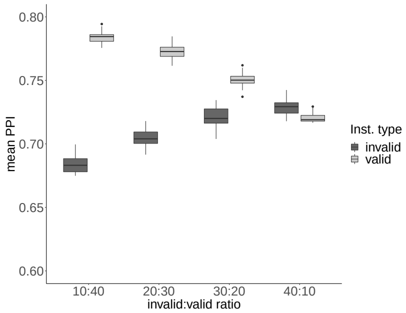

(d) full Bayesian

Figure S3: Box plots of PPI of truly valid and invalid instruments for balanced and directional pleiotropy (Scenario 2 and 4 respectively). On the x-axis is the ratio of invalid/pleiotropic and valid instruments, and the y-axis is the average PPI of 1,000 simulations. As shown by legend within plot, the different shades of grey denotes of the box plots denotes PPI of invalid and invalid instruments. (a) and (c) are the DL implementation, and (b) and (d) are the full Bayesian implementation.

E.4 Many weak instruments

Many weak instruments were simulated under scenario 1, but with 100 instruments. We experimented with 2 different mean F-statistics; 5 and 10. Table S4 gives the bias and coverage. Figure S4 shows PPI for valid and invalid instruments.

Table S4: Evaluation criteria with many weak instruments. 100 instruments in total. True β is 0.05. No. inv., Number of invalid instrument(s); Q, Q-statistics with exact weights; bias, mean bias; Cover., coverage; DL est., DL estimate; Full Bayes., Full Bayesian; $\bar{F}$, mean F-statistics.

**No. inv.**	**Q**	**IVW**		**DL est.**		**Full Bayes.**		**MR-APS**		**MR-RAPS**	
		Bias	Cover.	Bias	Cover.	Bias	Cover.	Bias	Cover.	Bias	Cover.
$\bar{\mathbf{F}} = 10$											
0	98.0	-0.019	9.30	-0.001	96.40	0.000	94.90	-0.000	93.70	0.000	93.10
20	109.3	-0.019	13.80	-0.001	96.30	0.003	92.90	0.003	90.10	0.002	91.50
40	120.2	-0.019	14.10	-0.000	96.70	0.006	89.50	0.007	84.10	0.006	85.90
60	131.0	-0.019	21.20	0.000	95.90	0.008	81.40	0.009	72.00	0.009	74.20
80	139.5	-0.019	21.10	0.000	96.40	0.012	73.20	0.012	62.10	0.011	65.10
100	149.4	-0.019	29.10	0.002	94.10	0.016	62.40	0.016	49.70	0.016	50.00
$\bar{\mathbf{F}} = 5$											
0	97.8	-0.038	0.00	-0.006	94.30	0.001	90.40	0.000	93.20	0.000	93.10
20	104.8	-0.039	0.00	-0.005	95.40	0.004	87.80	0.003	92.40	0.003	92.30
40	110.7	-0.038	0.00	-0.004	94.60	0.009	83.00	0.009	87.40	0.008	89.30
60	116.8	-0.038	0.00	-0.001	97.60	0.012	74.20	0.013	80.00	0.013	81.80
80	121.2	-0.038	0.00	0.001	96.30	0.016	68.90	0.015	74.50	0.016	74.40
100	125.7	-0.038	0.00	0.003	97.10	0.021	55.20	0.022	59.30	0.022	59.90

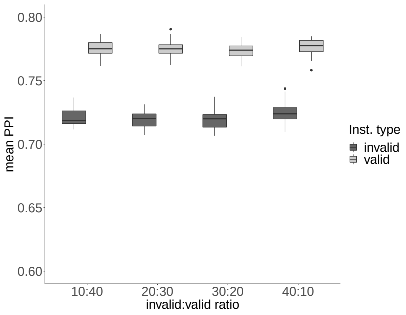

(a) $\bar{F}$=10: DL estimate

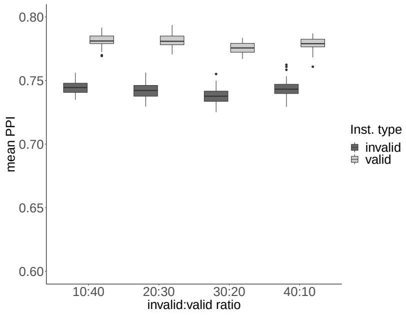

(b) $\bar{F}$=10: full Bayesian

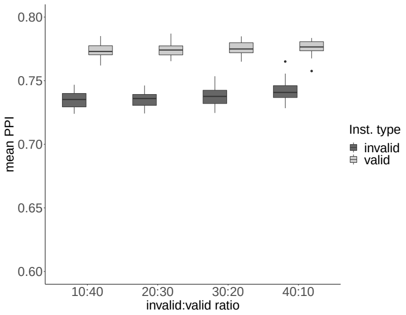

(c) $\bar{F}$=5: DL estimate

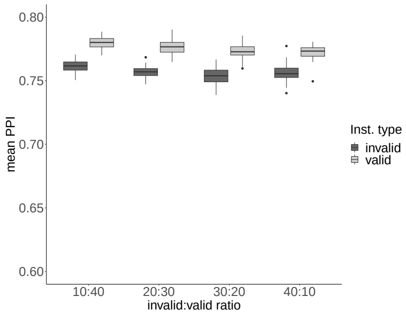

(d) $\bar{F}$=5: full Bayesian

Figure S4: Box plots of PPI of truly valid and invalid instruments for many weak instruments with mean F-statistics ($\bar{F}$) of 10 (a, b) and 5 (c, d). On the x-axis is the ratio of invalid/pleiotropic and valid instruments, and the y-axis is the average PPI of 1,000 simulations. As shown by legend within plot, the different shades of grey denotes of the box plots denotes PPI of invalid and invalid instruments. (a) and (c) are the DL implementation, and (b) and (d) are the full Bayesian implementation.

E.5 Non-normal pleiotropy

For assessing robustness to non-normal pleiotropy, pleiotropic effect was simulated from a long-tailed Student's t distribution with the same mean and variance as normally distributed pleiotropic effect. More specifically, Scenario 1 and 2 with $\alpha_j \sim t(5)/6^2$ instead. Table S5 gives the bias and coverage. Figure S5 shows the PPI for valid and invalid instruments.

Table S5: Evaluation criteria for nonnormal pleiotropic effect with 50 instruments in total. True β is 0.05. No. inv., Number of invalid instrument(s); Q, Q-statistics with exact weights; bias, mean bias; Cover., coverage; DL est., DL estimate; Full Bayes., Full Bayesian; $\bar{F}$, mean F-statistics.

**No. inv.**	**Q**	**IVW**		**DL est.**		**Full Bayes.**		**MR-APS**		**MR-RAPS**	
		Bias	Cover.	Bias	Cover.	Bias	Cover.	Bias	Cover.	Bias	Cover.
Scenario 1											
0	49.0	-0.001	96.40	-0.000	97.50	0.000	98.10	-0.000	94.40	-0.000	94.00
10	54.4	-0.000	94.70	-0.000	96.80	-0.000	97.10	-0.000	95.10	-0.000	95.10
20	60.0	-0.001	92.70	-0.000	97.10	-0.000	96.40	-0.001	94.40	-0.000	95.40
30	64.7	-0.001	91.60	0.000	96.40	0.000	95.80	0.000	94.40	-0.000	95.40
40	71.1	-0.001	89.90	0.001	95.00	0.001	93.90	-0.000	93.60	-0.000	94.00
50	75.5	-0.000	88.10	-0.000	93.50	-0.000	91.00	0.001	92.70	0.000	93.00
Scenario 2											
0	48.7	-0.018	33.40	-0.001	97.10	0.002	96.10	-0.000	93.90	-0.000	92.90
10	52.1	-0.018	38.20	-0.000	96.60	0.004	93.80	-0.002	95.80	-0.002	95.90
20	54.8	-0.018	37.90	0.001	98.40	0.006	93.20	-0.001	95.70	-0.001	96.60
30	58.0	-0.018	40.80	0.001	95.50	0.008	91.30	-0.001	94.40	-0.001	94.80
40	60.1	-0.019	40.80	-0.000	96.70	0.007	90.10	-0.001	94.50	-0.002	95.10
50	63.5	-0.019	44.80	0.001	95.20	0.010	83.90	-0.000	94.30	-0.000	94.30

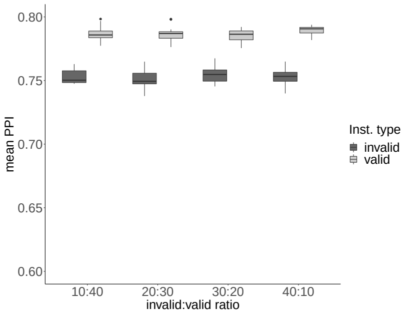

(a) Scenario 1: DL estimate

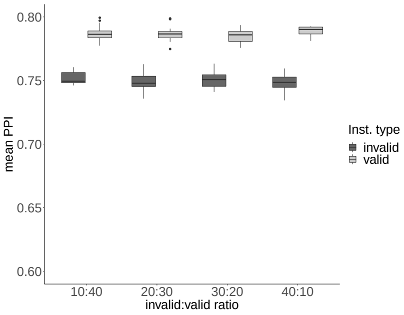

(b) Scenario 1: full Bayesian

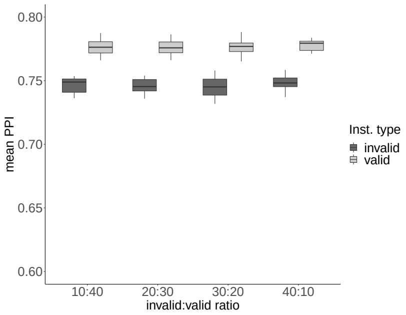

(c) Scenario 2: DL estimate

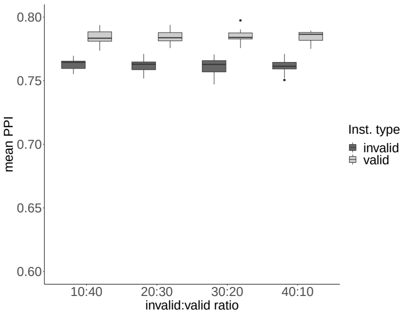

(d) Scenario 2: full Bayesian

Figure S5: Box plots of PPI of truly valid and invalid instruments for Scenario 1 and 2, where invalid instruments have nonnormal pleiotropic effect. On the x-axis is the ratio of invalid/pleiotropic and valid instruments, and the y-axis is the average PPI of 1,000 simulations. As shown by legend within plot, the different shades of grey denotes of the box plots denotes PPI of invalid and invalid instruments. (a) and (c) are the DL implementation, and (b) and (d) are the full Bayesian implementation.

E.6 Sensitivity to strengths of heterogeneity

We can use Q-statistics to monitor the heterogeneity between the causal effect estimate from each of the instruments [6]. This section investigates our approaches' sensitivity to the change in Q-statistics. Using Equation (9) and the χ^2 distribution for $L-1$ degrees of freedom, we could fix α_j^2 to give p-values for different levels of heterogeneity. We considered 2 forms of Q-statistics; (1) the true Q-statistics in total for 20% invalid instruments are 85, 75, 66 and 62 to give p-value of 0.001, 0.01, 0.05 and 0.1 respectively. (2) Each invalid instruments have true Q-statistics of 11, 7, 4 and 3 to give p-value of 0.001, 0.01, 0.05 and 0.1 respectively. But in total, it is borderline evidence for heterogeneity (Q-statistic p-value=0.05), hence, the number of invalid instruments increases with the Q-statistics. See Table S6 for a summary.

Table S6: Summary of Q-statistics (Q) simulation. The p-value for overall and individual Q is from χ^2 distribution of $L-1$ and 1 degrees of freedom respectively. Total number of instruments is 50. Ind., individual.

**Scenario**	**No. invalid**	**Overall Q (p-value)**	**Individual Q (p-value)**
Sum Q	10	85 (0.001)	8.5 (0.01)
	10	75 (0.01)	7.5 (0.01)
	10	66 (0.05)	6.6 (0.01)
	10	62 (0.1)	6.2 (0.01)
Ind. Q	6	66 (0.05)	11 (0.001)
	10	66 (0.05)	7 (0.01)
	17	66 (0.05)	4 (0.05)
	25	66 (0.05)	3 (0.1)

Our results demonstrate three facts:

1. Increasing heterogeneity with same number of invalid instruments does not affect the overall performance of the estimators, but only the inclusion probability of the instruments.

2. Increasing the number of invalid instruments whilst fixing the total heterogeneity does not affect the overall performance of the estimators.

3. When the pleiotropy parameters are small and exchangeable, the probability of inclusion is approximately constant across SNPs

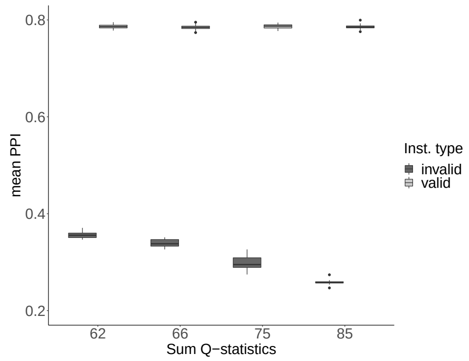

(a) Sum Q: DL estimate

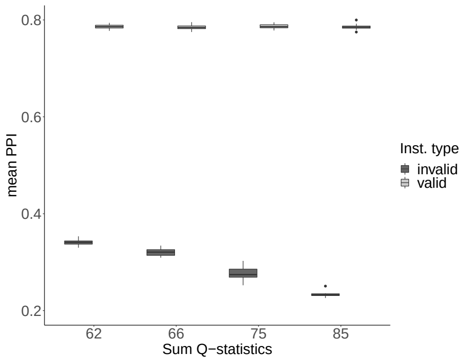

(b) Sum Q: full Bayesian

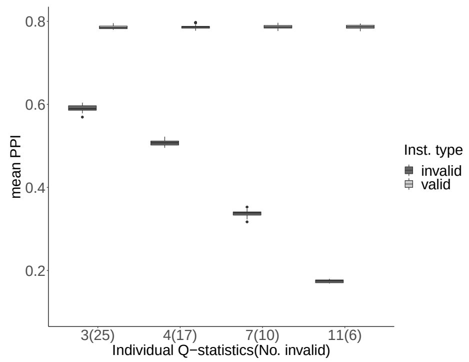

(c) Individual Q: DL estimate

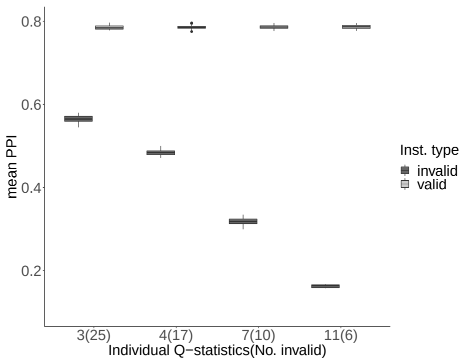

(d) Individual Q: full Bayesian

Figure S6: The box plots of PPI for (a, b) sum Q of all invalid instruments (c, d) when the fixed amount of heterogeneity (Q=66) is due to many weakly pleiotropic or a small number of highly pleiotropic SNPs. The y-axis is the average PPI of 1,000 simulations. As shown by legend within plot, the different shades of grey denotes of the box plots denotes PPI of invalid and invalid instruments. (a) and (c) are the DL implementation, and (b) and (d) are the full Bayesian implementation.

Table S7: Evaluation criteria for varying Q-statistics. 50 instruments with mean F-statistics of 100. True β is 0.05. No. inv., Number of invalid instrument(s); Q exact, estimated Q-statistics for all instruments with exact weights; Cover., coverage; Ind., individual.

**Scenario**	**No. inv.**	**Overall Q**	**Ind. Q**	**Q exact**	**DL est.**		**Full Bayes.**	
					Bias	Cover.	Bias	Cover.
Sum Q	10	85	8.5	134.5	0.001	94.90	0.001	92.30
	10	75	7.5	124.2	0.000	97.00	0.000	94.40
	10	66	6.6	115.3	0.001	96.10	0.001	93.60
	10	62	6.2	111.0	0.000	96.60	0.001	94.90
Ind. Q	6	66	11	113.8	0.000	96.80	0.000	95.90
	10	66	7	115.4	0.001	96.60	0.001	94.00
	17	66	4	113.7	-0.001	95.90	0.000	91.70
	25	66	3	116.7	-0.001	96.00	0.000	87.60

F Modified Metropolis-Hastings algorithm for InSIDE violating pleiotropy

The updating algorithm for β_1, β_2, τ_1^2 and τ_2^2 is the same as β and τ^2 in the one-component model respectively (Appendix C).

- **Update** I_1

  1. Generate a random number between 1 and L, define it as I_{1q}^* from $P(I_L)$, which is the qth element of I_1^*
  2. Set $I_{1d}^* = I_{i1d}$ for all $d \neq q$, if $I_{i2q} \neq 1$, otherwise repeat step 1.
  3. Set $I_{1q}^* = (I_{i1q} - 1)^2$.
  4. If $\sum_{j=1}^{L} I_{1j} \geq 5$ is true, proceed to next step, otherwise repeat step 1.
  5. Accept $I_{1i+1} = I_1^*$ with probability:

  $$prob = \min\left\{1, \frac{P(\beta_{1i+1}, \tau_{1i+1}^2, \beta_{2i+1}, \tau_{2i+1}^2, I_1^*, I_{2i})}{P(\beta_{1i+1}, \tau_{1i+1}^2, \beta_{2i+1}, \tau_{2i+1}^2, I_{1i}, I_{2i})}\right\}$$

  otherwise set $I_{1i+1} = I_{1i}$.

- **Update** I_2

  1. Generate a random number between 1 and L, define it as I_{2q}^* from $P(I_L)$, which is the qth element of I_2^*
  2. Set $I_{2d}^* = I_{i2d}$ for all $d \neq q$, if $I_{i1q} \neq 1$, otherwise repeat step 1.
  3. Set $I_{2q}^* = (I_{i2q} - 1)^2$.
  4. If $\sum_{j=1}^{L} I_{2j} \geq 5$ is true, proceed to next step, otherwise repeat step 1.
  5. Accept $I_{2i+1} = I_2^*$ with probability:

  $$prob = \min\left\{1, \frac{P(\beta_{1i+1}, \tau_{1i+1}^2, \beta_{2i+1}, \tau_{2i+1}^2, I_{1i+1}, I_2^*)}{P(\beta_{1i+1}, \tau_{1i+1}^2, \beta_{2i+1}, \tau_{2i+1}^2, I_{1i+1}, I_{2i})}\right\}$$

  otherwise set $I_{2i+1} = I_{2i}$.

Step 2 in **Update** I_1 and **Update** I_2 restricts the new jump to be conditional on I_2 and I_1 respectively, this will stop the case of $(I_{1j} = 1, I_{2j} = 1)$. Model space including both $(I_{1j} = 1, I_{2j} = 1)$ and $(I_{1j} = 0, I_{2j} = 0)$ is equivalent to giving model that consists of outlying instruments higher probability then models where instruments have to be designated to either I_1 or I_2.

G Simulations under the two-component model

This section is for two-component BESIDE-MR, that covers Monte Carlo simulation method, results for weaker instruments (L=50 and mean F-statistics of 10), and simulated example to demonstrate when a SNP belongs to S_0 (neither I_1 or I_2 clusters).

G.1 Simulation Method

Using the same underlying data generating Model (3) in the main manuscript, suppose that we have two different groups of invalid instruments: in the first group, S_1 we have $\psi_j = 0$ for all SNPs and $\bar{\upsilon} = \bar{\alpha} = 0$, shown in Appendix E. That is, the SNPs in S_1 exhibit balanced pleiotropy under the InSIDE assumption. For illustrative purposes, suppose now that the remaining instruments are in a set S_2, defined by $\delta_j = 0$, $\upsilon_j = 0$ and $\kappa_x = \kappa_y = 1$, but $\psi_j \neq 0$ have Uniform $U(0.34, 1.1)$ distribution. This means that that $\alpha_j = \gamma_j = \psi_j$, so that the InSIDE assumption is perfectly violated. Using the bias formulae, Equation (2.4) in the main manuscript, it follows that

$$\text{For } j \in S_1 : \hat{\Gamma}_j = \alpha_j + \beta\gamma_j + \sigma_{Yj}\epsilon_j$$
$$\text{For } j \in S_2 : \hat{\Gamma}_j = \alpha_j + \beta^*\gamma_j + \sigma_{Yj}\epsilon_j$$

where $\beta^* = \beta + 1$. The set of SNPs in S_2 therefore identify a distinct, biased version of the causal effect. In the general case where the SNPs could be classified into an InSIDE-respecting set and an InSIDE-violating set, it would be more reasonable to assume that α_j, γ_j and υ_j could all be non-zero. Although InSIDE would not then be maximally violated in S_2 we would still see two clusters in the data, albeit with a less defined separation.

The same evaluation criteria is used as for the one-component model but now PPI_{S_1} and PPI_{S_2} is probability of inclusion for S_1 and S_2 SNPs, where their numbers add up to L.

G.2 Scenario 6

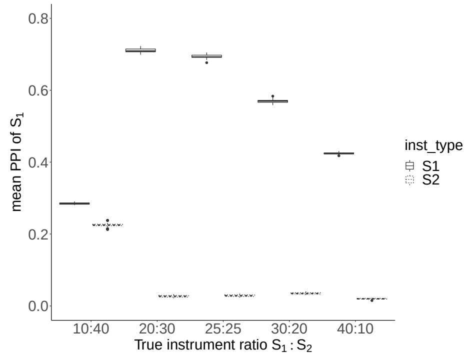

(a) DL estimate: PPI for S_1

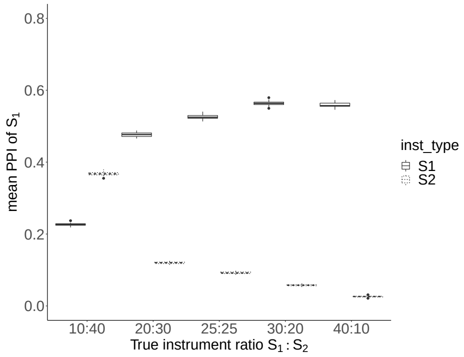

(b) full Bayesian: PPI for S_1

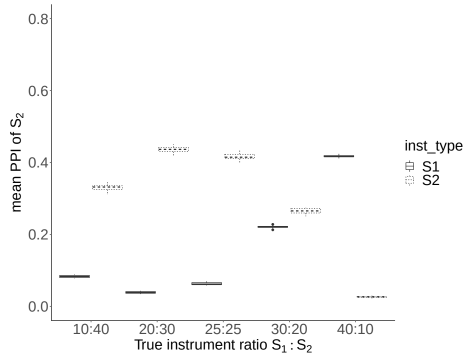

(c) DL estimate: PPI for S_2

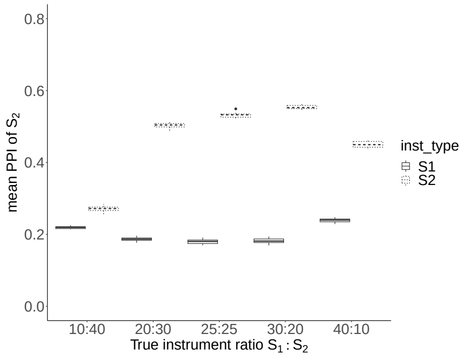

(d) full Bayesian: PPI for S_2

Figure S7: Box plots of PPI_{S_1} and PPI_{S_2} for truly S_1 and S_2 instruments in Scenario 6. The x-axis is the true ratio of instruments in each cluster (S_1:S_2), and the y-axis is the average PPI_{S_1} and PPI_{S_2} of 1,000 simulations. As shown by legend within plot, the different shades of grey of the box plots denotes PPI for truly S_1 and S_2 instruments. (a) and (c) are the DL implementation, and (b) and (d) are the full Bayesian implementation.

G.3 Weak instruments

We reduced the strength of instrument of scenario 6 to have mean F-statistics of 10; σ_{Xj} are generated from a Uniform $U(0.06, 1)$ distribution for both S_1 and S_2. Table S8 gives the bias and coverage. Figure S8 shows the PPI for S_1 and S_2 instruments.

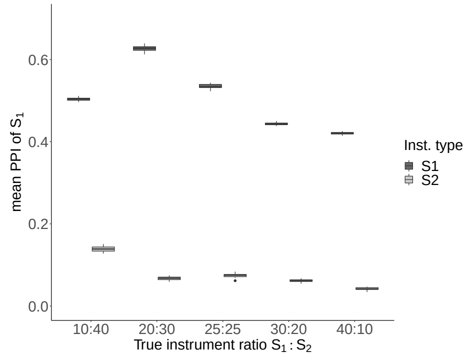

(a) DL estimate: PPI for S_1

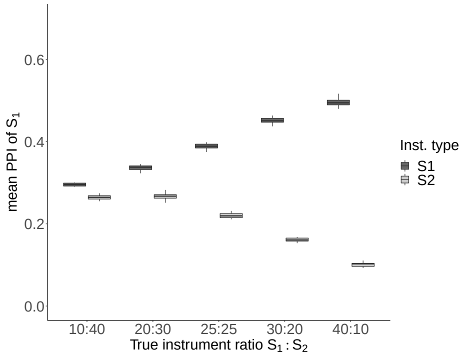

(b) full Bayesian: PPI for S_1

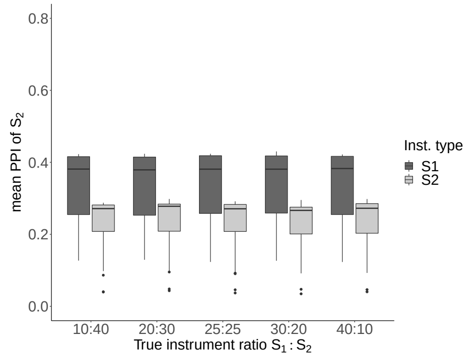

(c) DL estimate: PPI for S_2

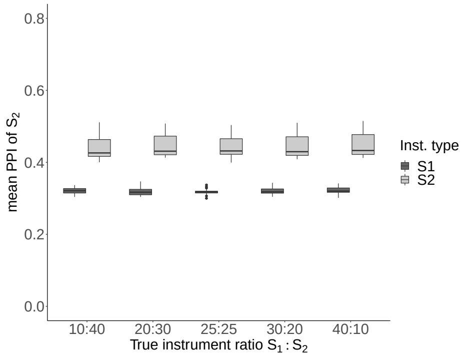

(d) full Bayesian: PPI for S_2

Figure S8: Box plots of PPI_{S_1} and PPI_{S_2} for truly S_1 and S_2 instruments in weak instruments (mean F-statistic of 10). The x-axis is the true ratio of instruments in each cluster (S_1:S_2), and the y-axis is the average PPI_{S_1} and PPI_{S_2} of 1,000 simulations. As shown by legend within plot, the different shades of grey of the box plots denotes PPI for truly S_1 and S_2 instruments. (a) and (c) are the DL implementation, and (b) and (d) are the full Bayesian implementation.

Table S8: Evaluation criteria for estimating two causal parameter from instruments with mean F-statistic of 10. 50 instruments in total. The true β is 0.05. Est., estimator; Inst., instrument(s); Q, exact Q-statistics; DL est., DL estimate; Full Bayes., Full Bayesian. β_1 is estimating β and β_2 for $\beta + 1$.

**Est.**	**Inst.**	**Q**		**mean bias**		**median bias**		**coverage**	
	$\mathbf{S_1 : S_2}$	$\mathbf{S_1}$	$\mathbf{S_2}$	β_1	β_2	β_1	β_2	β_1	β_2
	40:10	58.8	51.2	-0.004	-0.988	-0.003	-0.990	98.7	0.2
	30:20	43.5	118.9	0.026	-0.870	0.014	-0.974	98.4	18.3
DL est.	25:25	35.4	153.2	0.051	-0.532	0.020	-0.511	95.9	60.4
	20:30	28.0	205.5	0.027	-0.252	0.012	-0.185	94.8	86.8
	10:40	12.8	278.5	0.252	-0.143	0.178	-0.118	75.3	93.9
	40:10	58.8	51.2	-0.236	-0.478	-0.002	-0.050	81.4	70.3
	30:20	43.5	118.9	-0.395	-0.364	-0.009	0.064	65.9	69.8
Full Bayes.	25:25	35.4	153.2	0.551	-1.420	0.973	-1.025	30.1	41.4
	20:30	28.0	205.5	0.500	-1.566	1.062	-1.816	19.0	42.4
	10:40	12.8	278.5	0.258	-2.332	1.087	-3.133	1.9	27.2

G.4 Simulated example for S_0

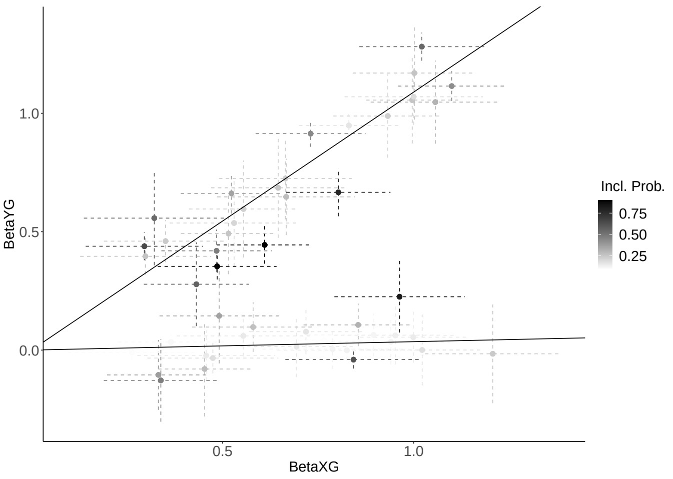

Figure S9: An association plot of a simulated example to demonstrate when a SNP is in S_0 (neither S_1 or S_2). The simulated S_1:S_2 ratio is 50:50 for strong instruments (mean F-statistic of 100). The 2 solid lines are the DL estimated effect sizes for the 2 clusters. As shown by legend; the colour gradient is the PPI for a instrument belonging to S_0, i.e. the darker the colour the higher the probability that the SNP belongs to S_0.

H Applied example

This section gives the PPI from two-component BESIDE-MR for each SNP. And results from sensitivity analysis for both one- and two-component BESIDE-MR.

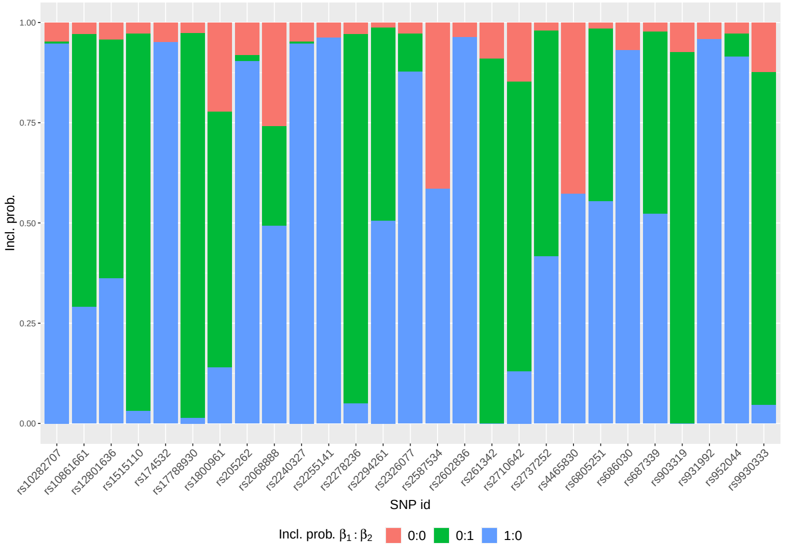

(a) DL estimate

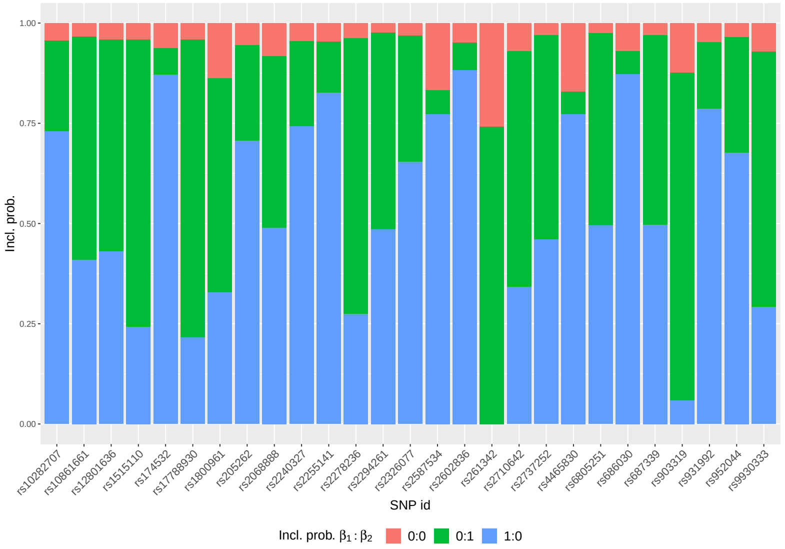

(b) Full Bayesian

Figure S10: AMD and HDL: PPI for DL estimate (a) and full Bayesian approach (b), assuming InSIDE violation. As shown by legend; colour red, green and blue is for instrument in neither (0:0), instrument estimating β_2 (0:1) and β_1 (1:0) respectively.

Table S9: Sensitivity analysis for one-component BESIDE-MR with non-zero penalisation term, η. Med., LCI and UCI are the median of the posterior distribution with 95% upper and lower credible intervals respectively. $\hat{\mathbf{Q}}$ is instrument normalised Q-statistics, $\sum \mathbf{Q_j}/\mathbf{I_j}$. $\sum I_j$ is the number of instruments included. The Q-statistic for 27 Instruments is 115.99.

η		**2**			**3**			**4**			**5**		
**Para.**	**Est.**	**Med.**	**LCI**	**UCI**	**Med.**	**LCI**	**UCI**	**Med.**	**LCI**	**UCI**	**Med.**	**LCI**	**UCI**
β	DL	0.78	0.53	1.08	0.79	0.53	1.08	0.78	0.53	1.07	0.78	0.53	1.07
	Bayes	0.77	0.50	1.08	0.77	0.50	1.07	0.77	0.50	1.07	0.76	0.49	1.07
$\tau^2 \times 10^{-4}$	DL	0.00	0.00	0.00	0.00	0.00	0.00	0.00	0.00	0.00	0.00	0.00	0.00
	Bayes	0.28	0.09	1.77	0.29	0.09	1.83	0.29	0.09	1.86	0.29	0.09	1.87
$\hat{\mathbf{Q}}$	DL	0.98	0.89	0.99	0.98	0.93	0.99	0.99	0.94	0.99	0.99	0.94	0.99
	Bayes	1.86	1.51	1.94	1.86	1.57	1.90	1.86	1.57	1.86	1.86	1.86	1.86
$\sum I_j$	DL	20	19	20	20	19	20	20	20	20	20	19	20
	Bayes	26	24	26	26	25	26	26	25	26	26	25	26

Table S10: Sensitivity analysis for two-component model with non-zero penalisation terms, η_1 and η_2. Median, 95% LCI and 95% UCI are the median of the posterior distribution with 95% upper and lower credible intervals respectively. $\hat{\mathbf{Q}}_1$ and $\hat{\mathbf{Q}}_2$ are the instrument normalised Q-statistics, $\sum \mathbf{Q_{1j}}/\mathbf{I_{1j}}$ and $\sum \mathbf{Q_{2j}}/\mathbf{I_{2j}}$ respectively. $\sum I_{1j}$ and $\sum I_{2j}$ are the number of instruments included in the 2 clusters. The Q-statistic for 27 Instruments is 115.99.

$\|\eta_1 - \eta_2\|$		**1**			**2**			**3**			**4**			**5**		
**Para.**	**Est.**	**Med.**	**LCI**	**UCI**	**Med.**	**LCI**	**UCI**	**Med.**	**LCI**	**UCI**	**Med.**	**LCI**	**UCI**	**Med.**	**LCI**	**UCI**
β_1	DL	0.91	-1.03	1.84	0.96	-0.77	1.86	0.93	0.57	1.67	0.93	0.54	1.67	0.89	0.58	1.50
	Bayes	0.86	-0.85	1.84	0.87	-0.72	1.82	0.86	0.42	1.57	0.86	0.43	1.58	0.84	0.52	1.36
β_2	DL	-0.52	-1.72	1.75	-0.67	-1.83	1.52	-0.79	-1.86	0.49	-0.78	-1.87	1.00	-0.82	-1.83	-0.04
	Bayes	0.43	-1.61	1.90	0.21	-1.71	1.89	-0.53	-1.80	1.75	-0.56	-1.82	1.74	-0.74	-1.79	1.49
$\tau_1^2 \times 10^{-4}$	DL	0.00	0.00	0.00	0.00	0.00	0.00	0.00	0.00	0.00	0.00	0.00	0.00	0.00	0.00	0.00
	Bayes	0.24	0.08	1.30	0.24	0.08	1.26	0.24	0.08	1.24	0.24	0.08	1.26	0.24	0.08	1.29
$\tau_2^2 \times 10^{-4}$	DL	0.00	0.00	0.00	0.00	0.00	0.00	0.00	0.00	0.00	0.00	0.00	0.00	0.00	0.00	0.00
	Bayes	0.25	0.08	1.48	0.25	0.08	1.53	0.25	0.08	1.50	0.25	0.08	1.51	0.25	0.08	1.52
$\hat{\mathbf{Q}}_1$	DL	0.82	0.33	0.99	0.82	0.35	0.99	0.90	0.57	1.00	0.90	0.57	1.00	0.94	0.72	1.00
	Bayes	1.26	0.51	2.15	1.25	0.53	2.11	1.36	0.82	2.01	1.37	0.84	2.02	1.44	1.03	2.01
$\hat{\mathbf{Q}}_2$	DL	0.70	0.16	0.99	0.69	0.14	0.98	0.73	0.16	0.98	0.73	0.17	0.98	0.77	0.25	0.98
	Bayes	1.22	0.25	2.68	1.21	0.22	2.82	1.20	0.23	2.94	1.19	0.23	2.88	1.14	0.26	3.03
$\sum I_{1j}$	DL	11	6	15	11	7	15	15	11	18	15	11	18	17	14	19
	Bayes	11	7	16	12	7	16	16	12	20	16	12	20	19	16	22
$\sum I_{2j}$	DL	6	5	9	5	5	7	5	5	6	5	5	6	5	5	6
	Bayes	6	5	9	5	5	7	5	5	6	5	5	6	5	5	5

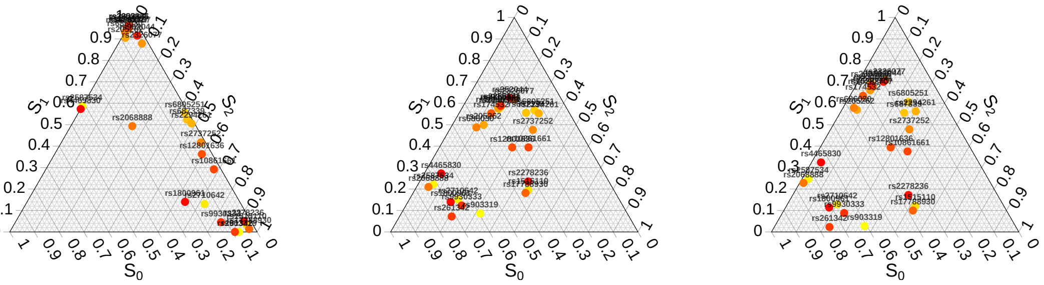

(a) $\eta_1 = \eta_2 = 0$ (b) $|\eta_1 - \eta_2| = 1$ (c) $|\eta_1 - \eta_2| = 2$

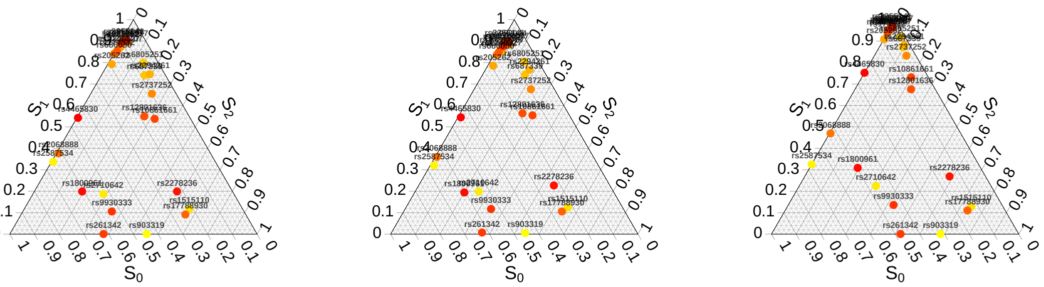

(d) $|\eta_1 - \eta_2| = 3$ (e) $|\eta_1 - \eta_2| = 4$ (f) $|\eta_1 - \eta_2| = 5$.

Figure S11: AMD and HDL: Ternary plot of η_1 and η_2 sensitivity on the inclusion probability for the two-component model with DL implementation. The colours represent different SNPs. The axes are inclusion probability for cluster S_1, S_2 and S_0.

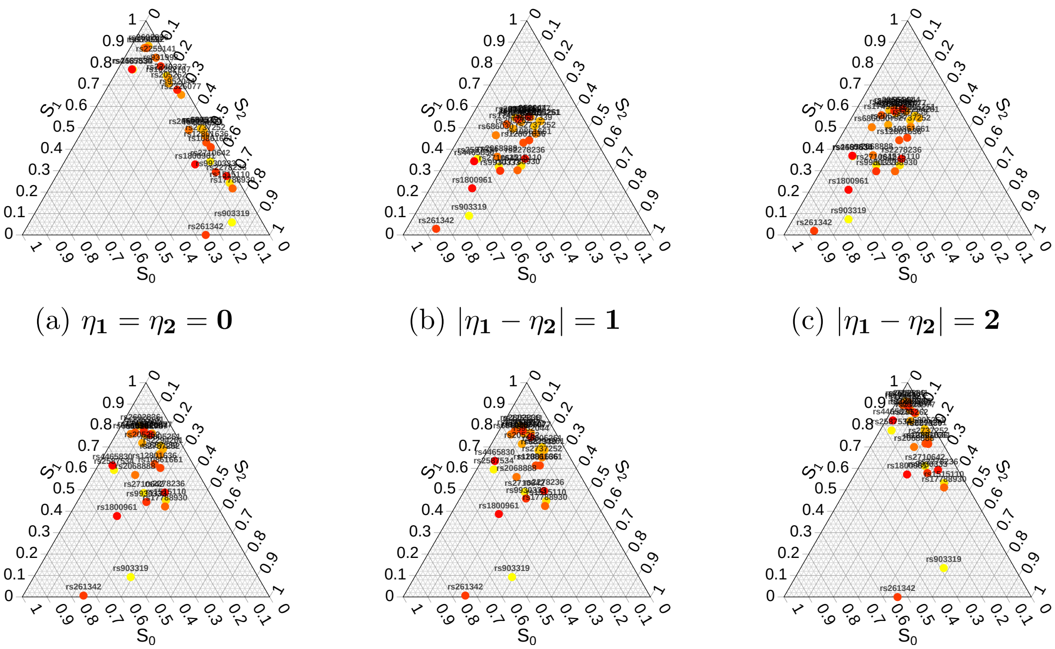

(a) $\eta_1 = \eta_2 = \mathbf{0}$ (b) $|\eta_1 - \eta_2| = \mathbf{1}$ (c) $|\eta_1 - \eta_2| = \mathbf{2}$

(d) $|\eta_1 - \eta_2| = \mathbf{3}$ (e) $|\eta_1 - \eta_2| = \mathbf{4}$ (f) $|\eta_1 - \eta_2| = \mathbf{5}$.

Figure S12: AMD and HDL: Ternary plot of η_1 and η_2 sensitivity on the inclusion probability for the two-component model with full Bayesian implementation. Colours are representing different SNPs. The colours represent different SNPs. The axes are inclusion probability for cluster S_1, S_2 and S_0.

References

[1] Jack Bowden, Fabiola Del Greco M, Cosetta Minelli, George Davey Smith, Nuala Sheehan, and John Thompson. A framework for the investigation of pleiotropy in two-sample summary data mendelian randomization. *Stat Med*, 36(11):1783–1802, 2017.

[2] Nuala A Sheehan and Vanessa Didelez. Epidemiology, genetic epidemiology and mendelian randomisation: more need than ever to attend to detail. *Human Genetics*, 139(1):121–136, 2020.

[3] Evan A Boyle, Yang I Li, and Jonathan K Pritchard. An expanded view of complex traits: from polygenic to omnigenic. *Cell*, 169(7):1177–1186, 2017.

[4] Jim Albert. *Bayesian computation with R*. Springer Science & Business Media, 2009.

[5] Rebecca DerSimonian and Nan Laird. Meta-analysis in clinical trials. *Controlled Clinical Trials*, 7(3):177–188, 1986.

[6] Jack Bowden, Fabiola Del Greco M, Cosetta Minelli, Qingyuan Zhao, Debbie A Lawlor, Nuala A Sheehan, John Thompson, and George Davey Smith. Improving the accuracy of two-sample summary-data Mendelian randomization: moving beyond the NOME assumption. *Int J Epidemiol*, 48(3):728–742, 12 2018.
